# Efficacy of dihydroartemisinin-piperaquine versus artemether-lumefantrine for the treatment of uncomplicated *Plasmodium falciparum* malaria among children in Africa: a systematic review and meta-analysis of randomized control trials

**DOI:** 10.1186/s12936-021-03873-1

**Published:** 2021-08-12

**Authors:** Dawit Getachew Assefa, Gizachew Yismaw, Eyasu Makonnen

**Affiliations:** 1 Center for Innovative Drug Development and Therapeutic Trials for Africa (CDT-Africa), College of Health Sciences, Addis Ababa University, Addis Ababa, Ethiopia; 2 Department of Nursing, College of Health Science and Medicine, Dilla University, Dilla, Ethiopia; 3 Department of Pharmacology and Clinical Pharmacy, College of Health Sciences, Addis Ababa University, Addis Ababa, Ethiopia

**Keywords:** Uncomplicated *Plasmodium falciparum*, Children, Randomized control trial, Artemisinin combination therapy, Dihydroartemisinin-piperaquine, Artemether-lumefantrine, Systematic review and meta-analysis, Africa

## Abstract

**Background:**

Emergence of *Plasmodium falciparum* resistance to artemisinin and its derivatives poses a threat to the global effort to control malaria. The emergence of anti-malarial resistance has become a great public health challenge and continues to be a leading threat to ongoing malaria control efforts. The aim of this review was to synthesize available evidence on the efficacy of dihydroartemisinin-piperaquine (DHA-PQ) compared to artemether-lumefantrine (AL) for the treatment of uncomplicated falciparum malaria among children in Africa.

**Methods:**

A systematic literature search was done to identify relevant articles from online databases PubMed/ MEDLINE, Embase, and Cochrane Central Register of Controlled Trials’ database (CENTRAL) for retrieving randomized control trials comparing efficacy of DHA-PQ and AL for treatment of uncomplicated falciparum malaria in African children. The search was performed from August 2020 to April 2021. Using Rev-Man software (V5.4.1), R-studio and Comprehensive Meta-analysis software version 3, the extracted data from eligible studies were pooled as risk ratio (RR) with 95% confidence interval (CI).

**Results:**

In this review, 25 studies which involved a total of 13,198 participants were included. PCR-unadjusted treatment failure in children aged between 6 months and 15 years was significantly lower in the DHA-PQ treatment arm on day 28 than that of AL (RR 0.14, 95% CI 0.08–0.26; participants = 1302; studies = 4; I^2^ = 0%, high quality of evidence). Consistently, the PCR-adjusted treatment failure was significantly lower with DHA-PQ treatment group on day 28 (RR 0.45, 95% CI 0.29–0.68; participants = 8508; studies = 16; I^2^ = 51%, high quality of evidence) and on day 42 (RR 0.60, 95% CI 0.47–0.78; participants = 5959; studies = 17; I^2^ = 0%, high quality of evidence). However, the efficacy was ≥ 95% in both treatment groups on day 28.

**Conclusion:**

From this review, it can be concluded that DHA-PQ reduces new infection and recrudescence on days 28 and 42 more than AL. This may trigger DHA-PQ to become a first-line treatment option.

**Supplementary Information:**

The online version contains supplementary material available at 10.1186/s12936-021-03873-1.

## Background

Malaria is the major cause of a vast majority of deaths among children under the age of five years [1–3]. In 2019, an estimated 229 million cases were reported globally from 87 malaria-endemic countries [3], of which 215 million cases were reported by the World Health Organization (WHO) African Region [3]. The risk of malaria infections among children aged under five years was higher in 2018, and the *Plasmodium falciparum* parasite was responsible for an estimated 24 million malaria cases in African children [1].

All African counties where falciparum malaria is endemic have introduced currently recommended artemisinin-based combination therapy (ACT) in confirmed cases of falciparum malaria since 2004 [1]. The artemisinin component is active against the sexual stages of the parasite that facilitates transmission to mosquitoes and covers two asexual cycles, and also rapidly decreases the number parasites by a factor of ~ 10,000 in each 48-h asexual cycle. The partner drug, with a longer half-life, eliminates the residual parasites over several weeks post-treatment, reducing repeated episodes and onward transmission, especially in high and seasonal transmission areas [4]. Artemisinin and partner drugs protect each other to prevent resistance development [5–8].

The efficacy of ACT has been excellent in Africa [9, 10]. Numerous trials have reported that dihydroartemisinin/piperaquine (DHA-PQ) is highly effective in treatment of uncomplicated falciparum malaria [11–15]. However, a previous review reported that prolongation of the QTc interval, pyrexia, early vomiting and diarrhoea, was common in patients treated with DHA-PQ [16]. The emergence of anti-malarial resistance has become a great public health challenge and continues to be a leading threat to ongoing malaria control efforts [17]. Resistance to ACT in Southeast Asia is becoming of highest concern [18]. There are a few reports on artemisinin resistance mediated by mutations *kelch13 (k13)* gene in the Greater Mekong Sub-region [19], Sudan [20], higher prevalence (42%) in Myanmar [21], and low frequency of *k13* gene mutation in 18 sub-Saharan African countries [22, 23]. In addition, over the past 10 years a decline in parasitological response in Nigeria [24], and a decrease in PCR-corrected therapeutic efficacy of ACT below 80% in Burkina Faso [25] has been noticed. Moreover, an increase in copy number of *plasmepsin* genes associated with decrease in effectiveness of piperaquine has arisen in Southeast Asia [26–28].

With the concern of resistance in Southeast Asia [26, 28–30], but with potential benefits of DHA-PQ over other artemisinin-based combinations [7, 31], it is necessary to assess if the treatment efficacy of this regimen in Africa has changed. Although several studies were conducted to assess the efficacy of ACT in adults yielding different success rates in Africa [32–34], there has been no systematic review and/or meta-analysis conducted to obtain strong evidence about the outcome of malaria treatment and artemisinin resistance in African children. The aim of this review was to synthesize available evidence on the efficacy of DHA-PQ compared to artemether-lumefantrine (AL) for the treatment of uncomplicated falciparum malaria among children in Africa in order to assist policymakers with appropriate national treatment policies and treatment protocols.

## Methods

This protocol has been registered at the International Prospective Register of Systematic Reviews (PROSPERO) database, ID: CRD42020200337 [35]. The methods and findings of the review have been reported according to preferred reporting for systematic reviews and meta-analyses (PRISMA 2020) [36].

### Eligibility criteria

The PICOS format was used to identify eligible studies [37].

### Participants

Children having uncomplicated falciparum malaria residing in Africa, regardless of gender, were included.

### Interventions

A target dose (range) of 4 (2–10) mg/kg body weight (bw) per day DHA and 18 (16–27) mg/kg bw per day PQ were given once a day for 3 days for children weighing ≥ 25 kg. The target dose (range) for children weighing < 25 kg of 4 (2.5–10) mg/kg bw per day DHA and 24 (20–32) mg/kg bw per day PQ once a day for 3 days.

### Comparator

The 1:6 fixed dose combination tablet consisting of artemether (20 mg) and lumefantrine (120 mg); the bw-adjusted dosages used were: 25–35 kg, 3 tablets per dose; 15–25 kg, 2 tablets per dose; and < 15 kg, 1 tablet. The medication was administered twice a day for 3 days (total 6 doses), the first two doses taken 8 h apart; the third dose taken 24 h after the first and then every 12 h on days 2 and 3**.**

### Primary outcomes

WHO methods and techniques for clinical trials on anti-malarial drug efficacy classification of genotyping to identify parasite populations was used to determine treatment outcome [38]. PCR genotyping to define treatment failure corresponding to current WHO recommendation was used [38].

#### PCR-unadjusted total failure

PCR-unadjusted total failure was calculated as the sum of late and early treatment failures (without PCR adjustment). The denominator excluded participants who did not satisfy inclusion criteria after randomization and those outcomes that were not available (e.g., lost to follow-up, withdrew consent, other species infection, took another anti-malarial, or failed to complete treatment).

#### PCR-adjusted total failure

Calculated PCR-adjusted total failure as the sum of early treatment failures plus late treatment failures due to PCR-confirmed recrudescence. Participants with indeterminate PCR results, missing PCR results or PCR-confirmed new infections were measured to be involuntary withdrawals and were excluded from the calculation. The denominator excluded participants who did not satisfy the inclusion criteria after randomization; participants with falciparum re-infection, other species mixed with falciparum re-infection, and undetermined or missing PCR; and, those for whom an outcome was not available (e.g., lost to follow-up, withdrew consent, other species infection, took another anti-malarial, or failed to complete treatment).

1. Polymerase chain reaction (PCR) confirmed parasitological failure by day 28 after starting treatment (defined as parasitaemia on any day between day 3 and 28, irrespective of clinical condition);
2. PCR-uncorrected parasitological failure by day 28 after starting treatment (defined as parasitaemia on any day between day 3 and 28, irrespective of clinical condition);
3. PCR-confirmed parasitological failure by day 42 after starting treatment (defined as parasitaemia on any day between day 3 and 42, irrespective of clinical condition);
4. PCR-uncorrected parasitological failure by day 42 after starting treatment (defined as parasitaemia on any day between day 3 and 42, irrespective of clinical condition);
5. PCR-confirmed parasitological failure for more than 42 days after starting treatment (defined as parasitaemia on any day between day 3 and 63, irrespective of clinical condition).
6. PCR-uncorrected parasitological failure for more than 42 days after starting treatment (defined as parasitaemia on any day between day 3 and 63, irrespective of clinical condition).

### Secondary outcomes

#### Fever clearance

The proportion of patients febrile on each day within 3 days.

#### Parasite clearance

The proportion of patients clear of parasites on each day within 3 days.

### Studies

Randomized controlled trials conducted in Africa which compared the efficacy of DHA-PQ *versus* AL for the treatment of uncomplicated falciparum malaria in children, written in English, and published between 2004 to April 2021 were included.

#### Electronic searches

A systematic literature search was done to identify relevant articles from online databases PubMed/ MEDLINE, Embase, and Cochrane Central Register of Controlled Trials’ database (CENTRAL). The search was limited to human trials, randomized control trials published between 2004 and April 2021. The search was done according to guidance provided in the Cochrane Handbook for Systematic Reviews of Interventions [37]. Additionally, ClinicalTrials.gov and the WHO International Clinical Trials Registry Platform, and the US Food and Drug Administration (FDA) were searched to assess ongoing or unpublished trials.

The search strategies in PubMed for the MeSH terms and text words was “Child” [Mesh]) AND “*Plasmodium falciparum*” [Mesh]) OR “Acute malaria” [Supplementary Concept]) OR “Artemether, Lumefantrine Drug Combination/therapeutic use” [Mesh]) OR “Lumefantrine” [Mesh]) OR “dihydroartemisinin” [Supplementary Concept]) OR “piperaquine” [Supplementary Concept]) OR (“Randomized Controlled Trial” [Publication Type] OR “Randomized Controlled Trials as Topic” [Mesh] OR “Controlled Clinical Trial” [Publication Type])) AND (“Drug Therapy” [Mesh] OR “Drug Therapy, Combination” [Mesh] OR “drug therapy” [Subheading])) AND (“Africa” [Mesh] OR “Africa South of the Sahara” [Mesh] OR “Africa, Western” [Mesh] OR “Africa, Southern” [Mesh] OR “Africa, Northern” [Mesh] OR “Africa, Eastern” [Mesh] OR “Africa, Central” [Mesh]. The searching strategies for CENTRAL and Embase are found in Additional file 1.

### Study selection, data collection, and data analysis

**Fig. 1:**
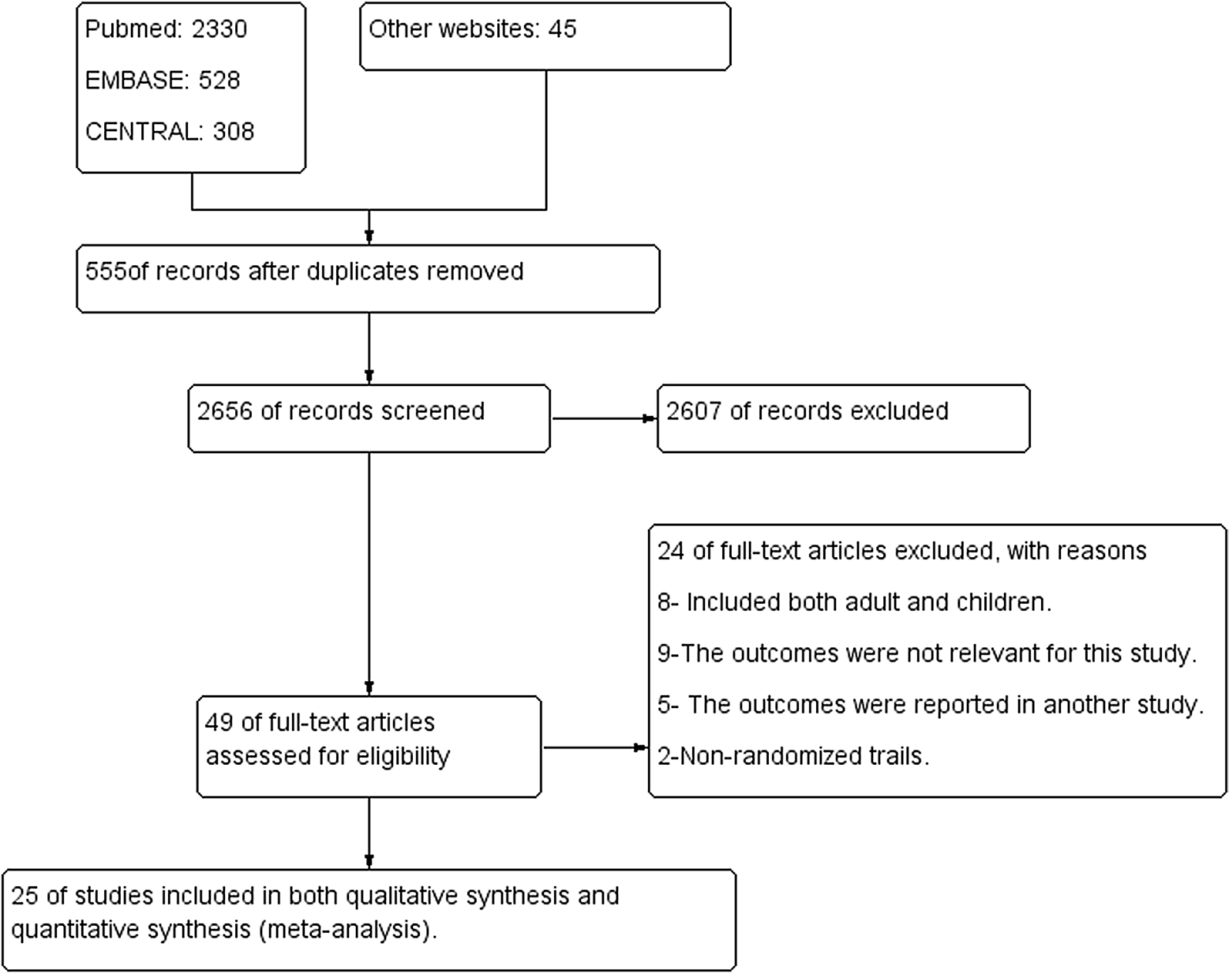
PRISMA study flow diagram of the study

The Cochrane Handbook for Systematic Reviews of Interventions [39] was followed. The software packages provided by Cochrane (RevMan 5.4), R-Studio and Comprehensive Meta-analysis version 3 were used. To import the research articles from electronic databases and remove duplicates, ENDNOTE software version X7 was used. Two authors independently reviewed the results of the literature search and obtained full-text copies of all potentially relevant trials. Disagreements were resolved through discussion. When clarification was necessary, the trial authors were contacted for further information. The screening and selection process was reported in a PRISMA flow chart (Fig. 1).

### Data extraction and management

Titles and abstracts from the electronic search were independently screened by two authors based on randomized control trials (RCTs) that assessed human falciparum malaria. The information collected were trial characteristics, including methods, participants, interventions, outcomes, data on dose, and drug ratios of the combinations. Relevant information such as title, journal, year of publication, publication status, study design, study setting, malaria transmission intensity, follow-up period, sample size, funding of trial or sources of support, baseline characteristics of study subjects, treatment failure, fever clearance, and parasite clearance were extracted from each article using an extraction format in the form of a table adapted from the Cochrane online training materials repository and modified for this study. The numbers of participants randomized and analyzed in each treatment group for each outcome were also collected. One author independently extracted data and the information collected was cross-checked by another investigator. Missing data were requested from authors whenever necessary. The numbers of participants experiencing the event and participating in each treatment group were documented.

### Assessment of risk of bias in included studies

The risk of bias for each trial was evaluated by two review authors independently using the Cochrane Collaboration’s tool for assessing the ‘risk of bias’ [37]. The risks were classified as high risk, unclear risk and low risk.

### Measures of treatment effect

The main outcomes in this review were total treatment failure on day 28, 42 and 63, PCR-adjusted and PCR-unadjusted. The numbers of patients with adequate clinical and parasitological response (ACPR) from the studies were combined and presented using risk rations accompanied by 95% confidence interval (CI).

### Unit of analysis issues

Participants were included according to the treatment group of the randomized clinical trials.

### Assessment of heterogeneity

Heterogeneity among the included trials was assessed by inspecting the forest plots (to detect overlapping CI) and the Cochrane Q and I^2^ statistic used to measure heterogeneity among the trials in each analysis, the Chi^2^ test with a P < 0.10 to indicate statistical significance, and the results were interpreted following Cochrane Handbook for Systematic Reviews of Interventions Version 6.0, Chapter 10: analysing data and undertaking meta-analyses [40].

- 0–40%: might not be important;
- 30–60%: may show moderate heterogeneity;
- 50–90%: may show substantial heterogeneity;
- 75–100%: considerable heterogeneity.

### Outlier and influence case analysis

To assess the distortion in the pooled effect estimate caused by one or more studies with extreme effect sizes, the pooled effect was checked again by removing outliers from the analysis [41]. To assess if the effect did not depend on one single study and whether there were studies that heavily pushed the effect of the analysis in one direction, influence case analysis was done [41]. To further investigate the contribution of each study to the overall heterogeneity of the meta-analysis, Baujat Plot was used [42].

### Graphic display of heterogeneity (GOSH) plot analysis

To further investigate the pattern of effect sizes and heterogeneity in the data, GOSH plot analysis was used. Using this plot, different sub-clusters with different effect size that were candidate for sub-group analysis were identified. When substantial heterogeneity (I^2^ > 50%) was identified, it was reported and possible causes by sub-group analyses were explored.

### Assessment of reporting bias

To assess the possibility of publication bias, funnel plots for asymmetry (Egger’s test P < 0.05) were used. When the Egger's test showed publication bias, the P-curve was used to estimate the presence of a “true” effect size behind the findings, and that the results were not the product of publication bias and P-hacking alone [43, 44]. To do the P-curve, R packages such as meta, demeter, stringr and poibin were used (Additional file 2).

### Data synthesis

The meta-analyses was done in consistency with the recommendations of Cochrane [39]. To aid interpretation, identity codes were given to include trials together with the first author, year of publication, and the 3 first letters of the country in which the trial was conducted. Trials were shown in forest plots in chronological order of the year the trials were published. A random-effects model was used, as trials were done by different researchers operating independently, and it could be implausible that all trials had functional equivalence, with a common effect estimate.

### Sub-group analysis and investigation of heterogeneity

The potential sources of heterogeneity were investigated through the following sub-group analyses: participants under 5 years old compared with participants between 6 months to 15 years old.

### Meta-regression

Meta-regression was used to investigate the association of study characteristics which cause heterogeneity with treatment effect. The covariates were age, HIV status, malaria transmission intensity in publication included, risk of bias, region, and drug administration (night dose). The results were presented in Figures and Tables.

### Sensitivity analysis

To investigate the strength of the methodology used in the primary analysis, a series of sensitivity analyses were conducted. To restore the integrity of the randomization process, the following steps were used: adding and excluding trials which were classified as high risk for bias back into the analysis in a stepwise fashion. To assess the influence of small-study effects on the results of the meta-analysis, fixed-effect and random-effect estimates of the intervention effect were compared. The robustness of the meta-analysis results explored the using influence analyses and the leave-one-out method.

### Quality of evidence

Quality of evidence was assessed using GRADE criteria and GRADE pro software [45]. The results were presented in a ‘Summary of Findings’ Table. Randomized trials are initially categorized as high quality but downgraded after assessment of five criteria [46]. The levels of evidence were defined as ‘high’, ‘moderate’, ‘low’, or ‘very low’. The recommendations of Sect. 8.5 and Chapter 13 of the Cochrane Handbook for Systematic Reviews of Interventions were followed [47]. The imprecision was judged based on the optimal information size criteria and CI [48].

## Results

A total of 3211 studies from databases were searched, of which 49 full-text trials for eligibility were assessed; 25 fulfilled the inclusion criteria for meta-analysis and for qualitative analysis (Fig. 1). The reason for excluding 24 studies is described in Additional file 3.

### Study characteristics

In this review, 25 studies were included, in which 13,198 participants with uncomplicated falciparum malaria were included (Additional file 4).

### Methodological quality and risk of bias

**Fig. 2:**
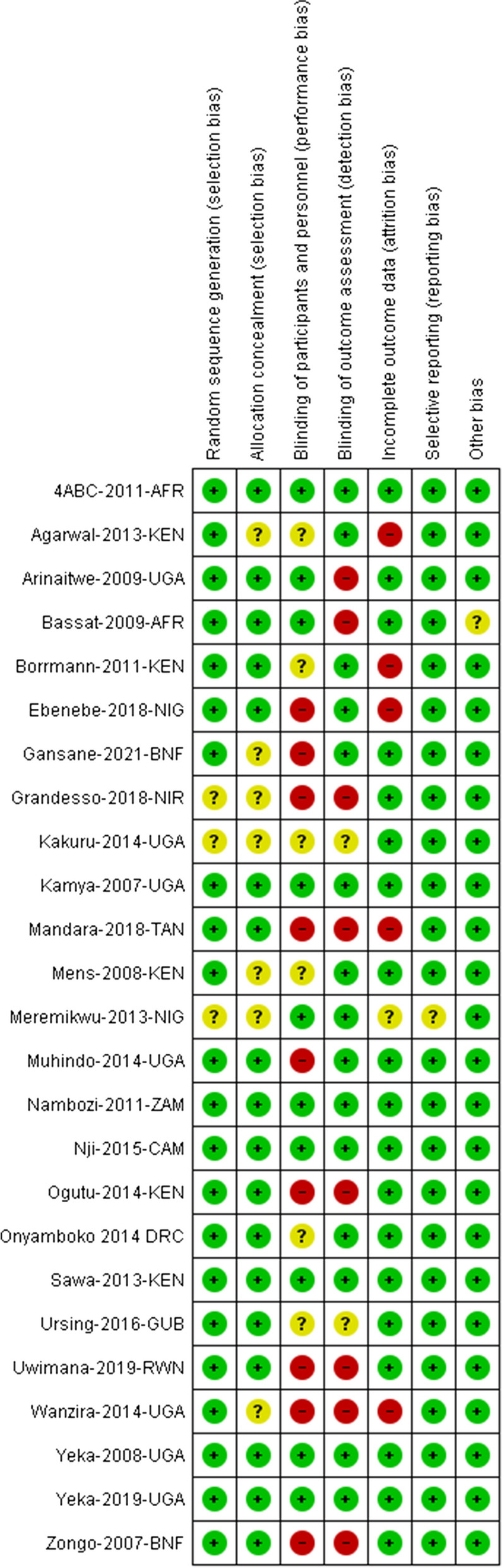
A summary of review authors' judgments about each risk of bias item for each included study

The risk of bias assessments is summarized in Fig. 2.

### Effect of interventions

#### PCR-unadjusted total failure on day 28

**Fig. 3:**
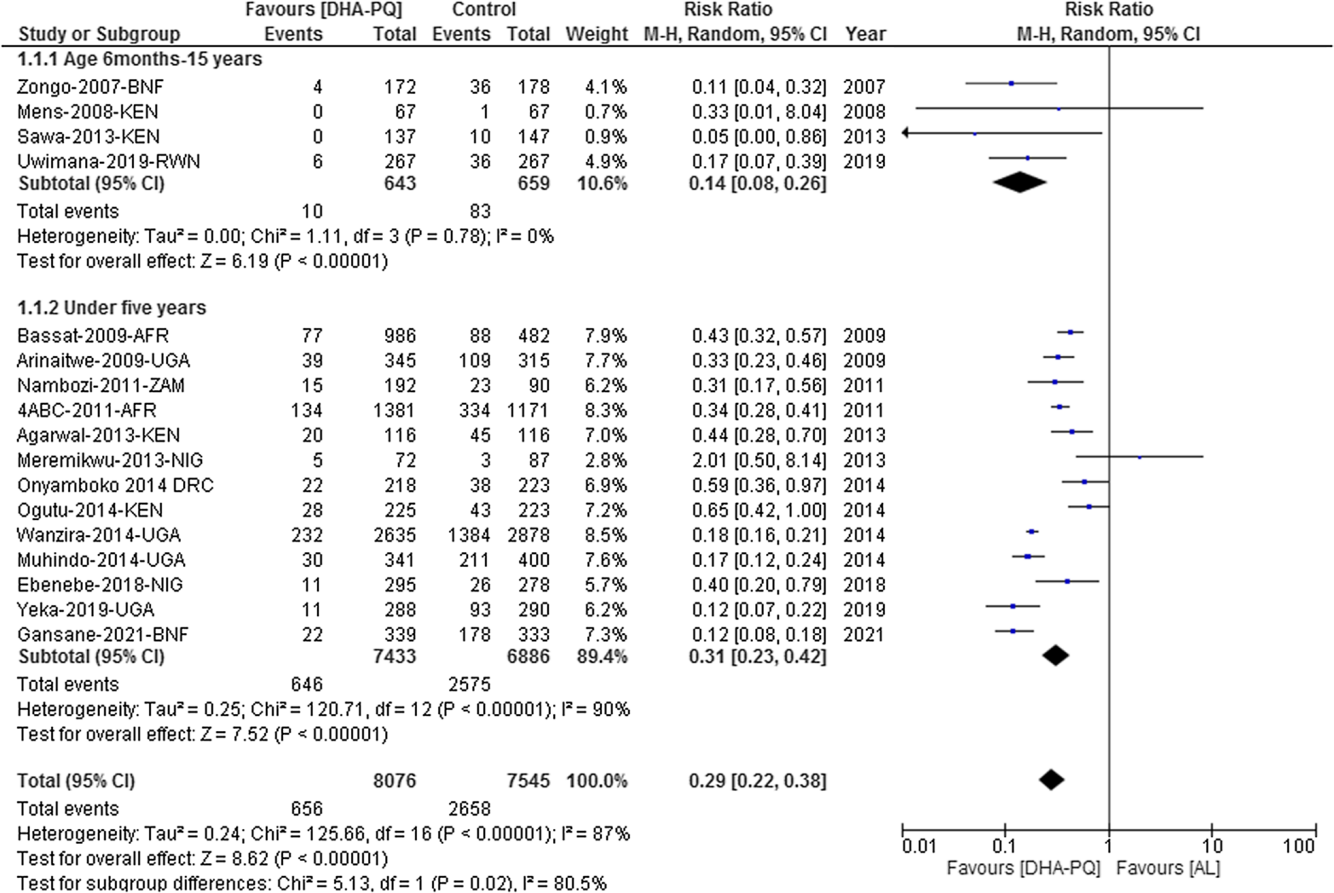
Forest plot of comparison between dihydroartemisinin-piperaquine and artemether-lumefantrine for treatment of uncomplicated falciparum malaria among children in Africa on PCR-unadjusted treatment failure on day 28

The PCR-unadjusted treatment failure in patients aged between 6 months and 15 years was lower in patients who were treated with DHA-PQ than that of AL [RR 0.14, 95% CI 0.08–0.26; participants = 1302; studies = 4; I^2^ = 0%, high quality of evidence (Fig. 3)]. However, the PCR-unadjusted treatment failure in children under five was heterogonous (Tau^2^ = 0.25; Chi^2^ = 120.71, df = 13 (P < 0.00001); I^2^ = 90%, moderate quality of evidence). The result could not be pooled. As there was high heterogeneity, it was more useful to consider the individual trial results. In 12/13 studies, the risk of treatment failure unadjusted by genotyping in children under five was significantly lower in the DHA-PQ treatment group than that of AL. Statistically significant difference was found between the two sub-groups [Chi^2^ = 5.13, df = 1 (P = 0.02); I^2^ = 80.5% (Fig. 3)].

### Meta-regression of day 28 PCR-unadjusted treatment failure

Based on the description of the malaria intensity in the included publications, studies were classified as very high, high, moderate, and low transmission intensity. Both age of the participants and malaria transmission intensity within the countries had a direct relationship with the relative risk of developing treatment failure unadjusted by genotyping [p = 0.034 and p = 0.024 (Additional file 5 and Additional file 6)]. The relative risk of developing treatment failure unadjusted by genotyping in under fives was higher by 8.5% compared to children age 6 months to 15 years, keeping malaria transmission intensity constant. The relative risk of developing treatment failure unadjusted by genotyping in children living in the area where malaria transmission intensity was moderate was higher by 42.2% compared to children living in high malaria transmission setting, keeping age of children constant (Table 1).

**Table 1:** Meta- regression of PCR-unadjusted treatment failure at day 28

Covariate	Coefficient	SE	95% Lower	95% Upper	Z-value	2-sided P-value
Intercept	− 2.3013	0.4315	− 3.1469	− 1.4556	− 5.33	0.0000
Age: under five	0.9137	0.4305	0.0699	1.7575	2.12	0.0338
Transmission: moderate	0.5776	0.2563	0.0752	1.0800	2.25	0.0242

Either the age of children or malaria transmission in the countries was associated with treatment failure (Q = 8.61, df = 2, P = 0.0135). The risk of treatment failure varied across the studies, meaning that some studies had high treatment failure and others had low treatment failure. Of the total variance in treatment failure, only 32% were explained by participant age and malaria transmission intensity within the countries. The funnel plot shows that all studies lay symmetrically around the pooled effect estimate (Egger’s test: intercept 0.98 (95% CI − 1.17, 3.13), P = 0.39 (Additional file 7).

#### PCR-adjusted total failure at day 28

**Fig. 4:**
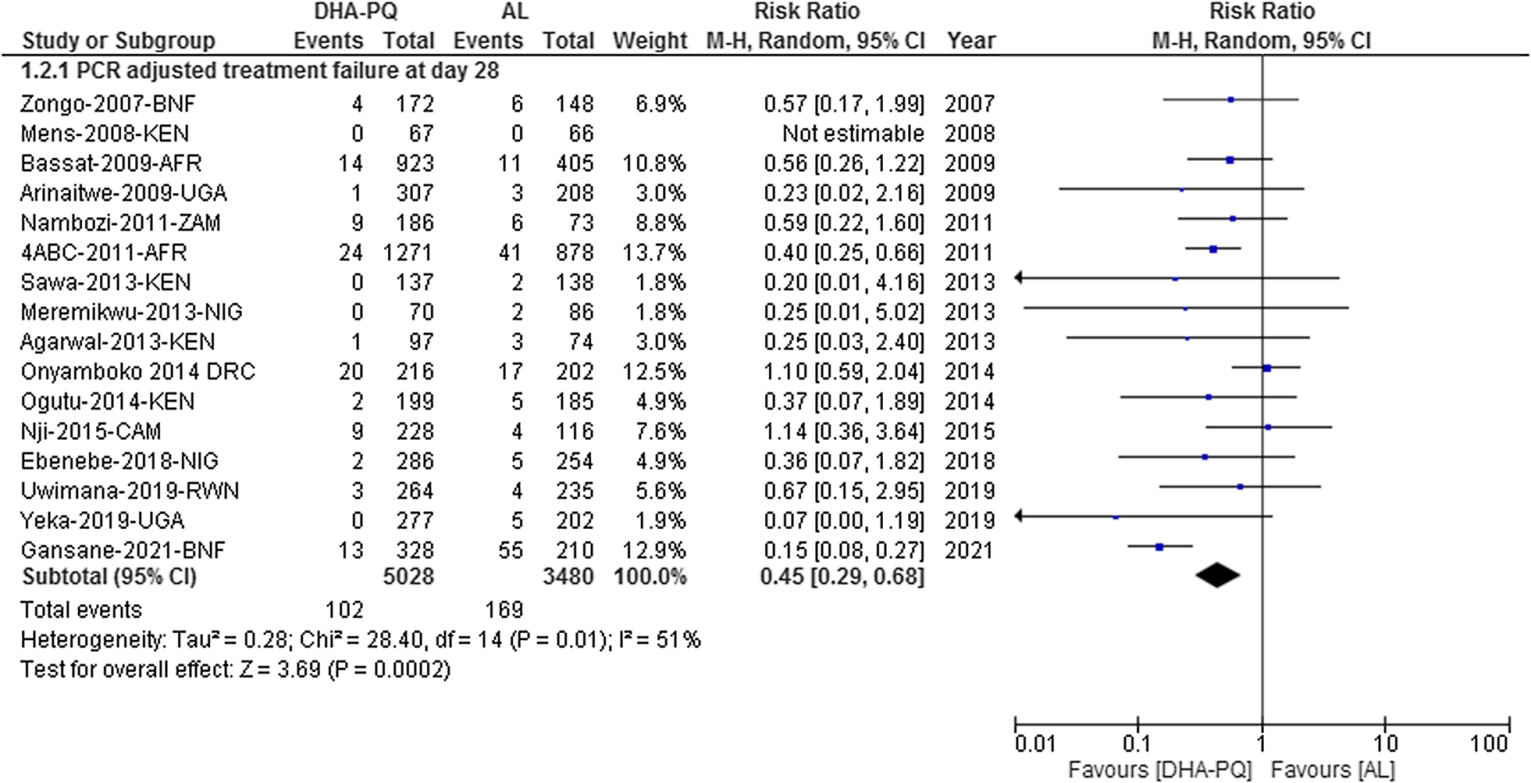
Forest plot of comparison between dihydroartemisinin-piperaquine and artemether-lumefantrine for treatment of uncomplicated falciparum malaria among children in Africa on PCR-adjusted treatment failure on day 28

On day 28, the PCR-adjusted treatment failure was significantly lower in DHA-PQ group than that of AL [RR 0.45, 95% CI 0.29–0.68; participants = 8508; studies = 16; I^2^ = 51%, high quality of evidence (Fig. 4)]. The funnel plot shows that all studies lay symmetrically around the pooled effect estimate implying that there was no publication bias (Egger’s test: − 0.19007 (95% CI − 1.77, 1.39), P = 0.799 (Additional file 8).

#### PCR-unadjusted total failure at day 42

**Fig. 5:**
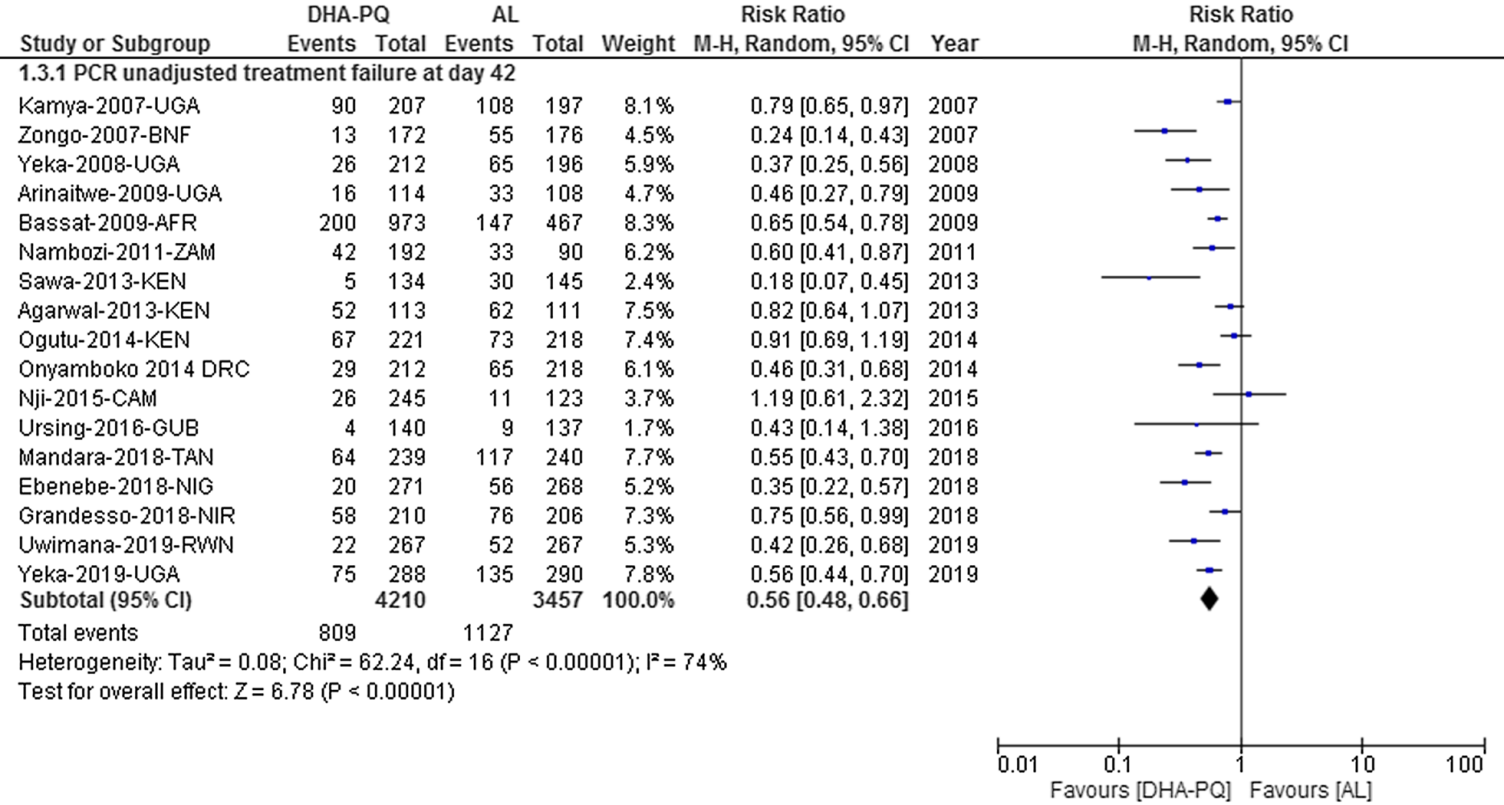
Forest plot of comparison between dihydroartemisinin-piperaquine and artemether-lumefantrine for treatment of uncomplicated falciparum malaria among children in Africa on PCR unadjusted treatment failure on day 42

The result had an unexplained considerable heterogeneity between the included studies [Tau^2^ = 0.08; Chi^2^ = 62.24, df = 16 (P < 0.00001); I^2^ = 74, moderate quality of evidence (Fig. 5)]. Considering individual study result, the PCR-unadjusted risk of recurrent falciparum parasitaemia in 13 studies was significantly lower in DHA-PQ group than AL. In four studies, however, the PCR-unadjusted risk of recurrent falciparum parasitaemia did have significant difference between the two treatment groups.

**Fig. 6:**
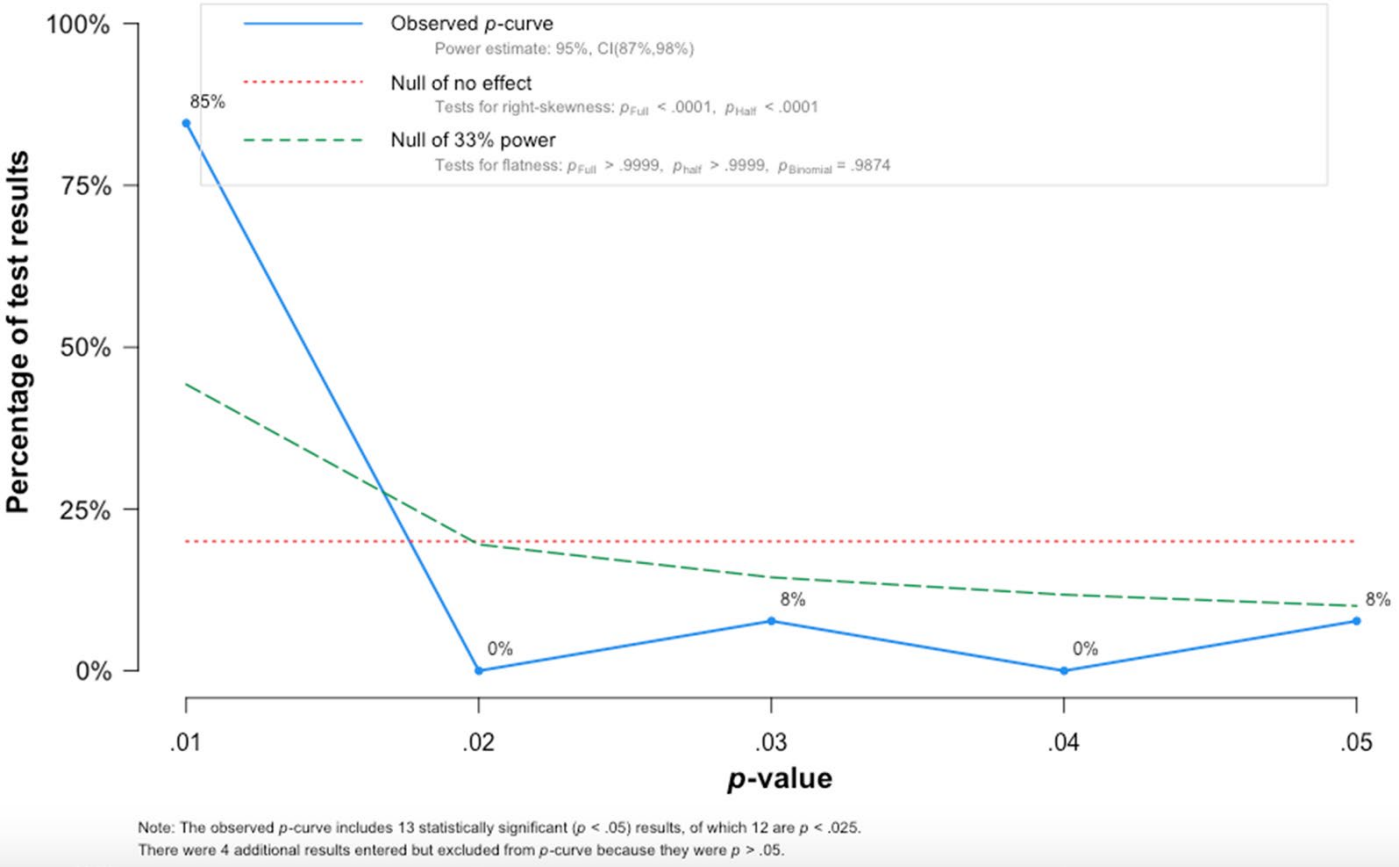
P-curve of comparison between dihydroartemisinin-piperaquine and artemether-lumefantrine for treatment of uncomplicated falciparum malaria among children in Africa on PCR unadjusted treatment failure on day 42

The funnel plot shows that all studies did not lie symmetrically around the pooled effect estimate implying that there was a publication bias (Egger’s test: − 2.48 (95% CI − 4.55, − 0.42), P = 0.021). The P-curve evaluation shows that 13 studies were included into the analysis, of which 12 had a P-value lower than 0.025. The power of the analysis was 95% (95% CI: 87%, 98%). The result shows that the evidential value was present, and there was a true effect size behind the findings, and that the results were not the product of publication bias and P-hacking alone (Fig. 6).

#### PCR-adjusted total failure at day 42

**Fig. 7:**
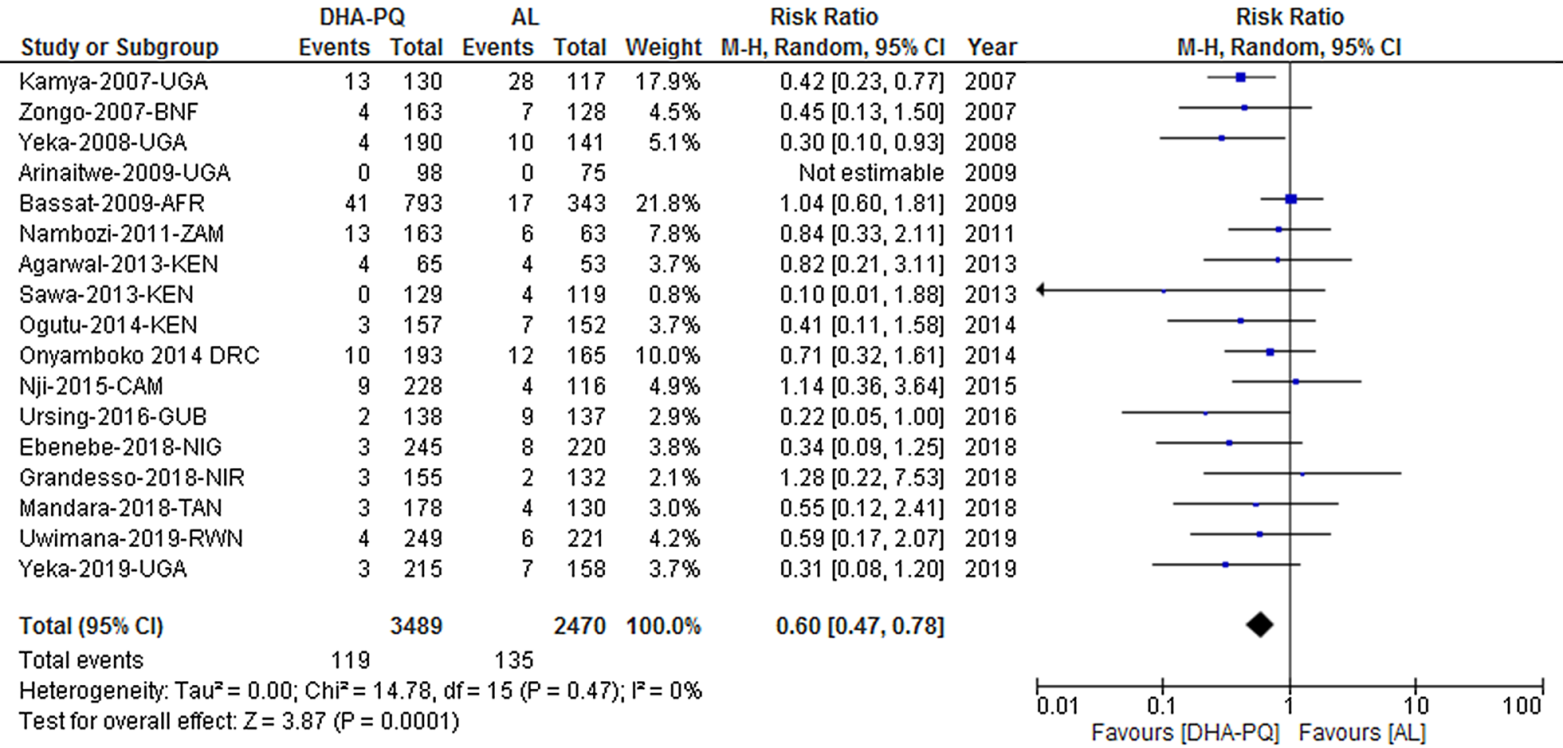
Forest plot of comparison between dihydroartemisinin-piperaquine and artemether-lumefantrine for treatment of uncomplicated falciparum malaria among children in Africa on PCR adjusted treatment failure on day 42

The PCR-adjusted treatment failure on day 42 was lower for participants treated with DHA-PQ than those treated with AL [RR 0.60, 95% CI 0.47–0.78; participants = 5959; studies = 17; I^2^ = 0%, high quality of evidence (Fig. 7)]. The funnel plot shows that all studies lay symmetrically around the pooled effect estimate implying that there was no publication bias (Egger’s test: − 1.06 (95% CI − 2.38, 0.25), p = 0.10 (Additional file 9).

#### PCR-unadjusted total failure at day 63

**Fig. 8:**
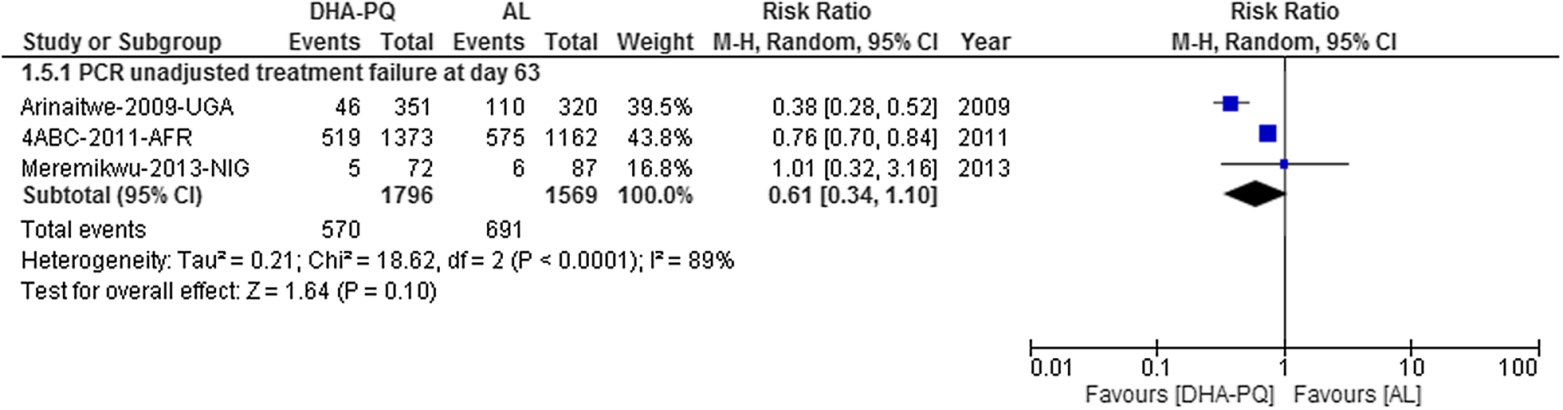
Forest plot of comparison between dihydroartemisinin-piperaquine and artemether-lumefantrine for treatment of uncomplicated falciparum malaria among children in Africa on PCR unadjusted treatment failure on day 63

Three studies with 3,365 participants were included in this analysis. The result had considerable heterogeneity (Tau^2^ = 0.21; Chi^2^ = 18.62, df = 2 (P < 0.0001); I^2^ = 89%, moderate quality of evidence) and the result could not be pooled. It is more useful to consider individual trial results. On day 63, the PCR-unadjusted treatment failure in participants treated with DHA-PQ was significantly lower than those treated with AL in the two studies. However, the relative risk of PCR-unadjusted treatment failure was not significantly different between the two treatment groups (Fig. 8).

#### PCR-adjusted total failure at day 63

**Fig. 9:**
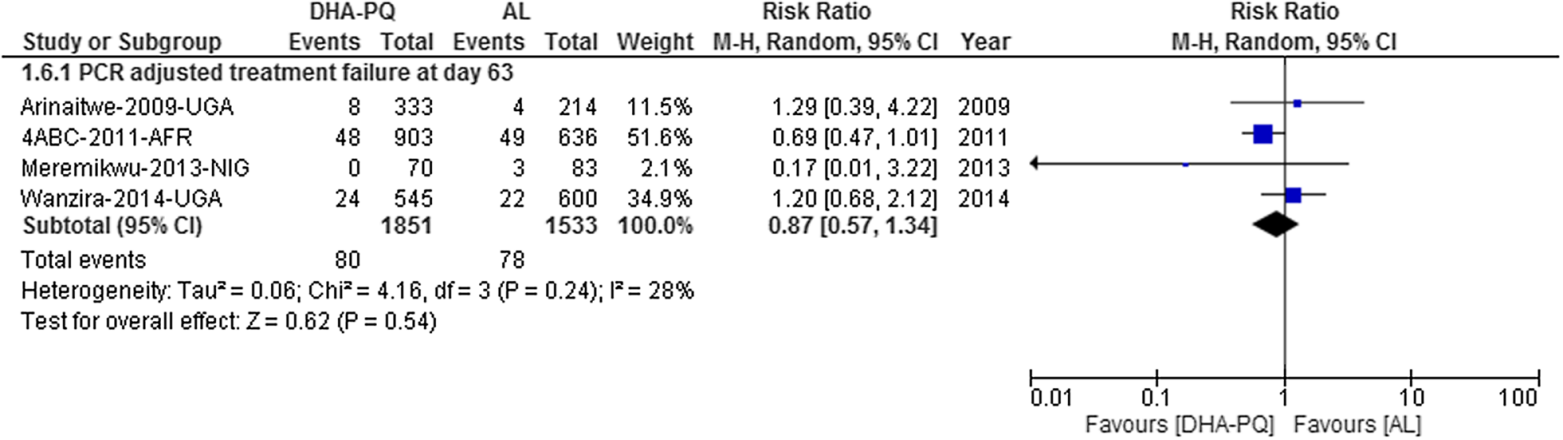
Forest plot of comparison between dihydroartemisinin-piperaquine and artemether-lumefantrine for treatment of uncomplicated falciparum malaria among children in Africa on PCR adjusted treatment failure on day 63

The PCR-adjusted treatment failure at day 63 was significantly lower in both treatment groups without statistically significant difference [RR 0.87, 95% CI 0.57–1.34; participants = 3384; studies = 4; I^2^ = 28%, high quality of evidence (Fig. 9)].

#### PCR-unadjusted and -adjusted treatment failure at day 84

Two studies had followed the patients up to 84 days. The PCR-adjusted treatment failure on day 84 was more than 10% in AL treatment group [49]. On day 84, the number of re-infections was the same in both treatment groups [49, 50].

### Fever clearance

**Fig. 10:**
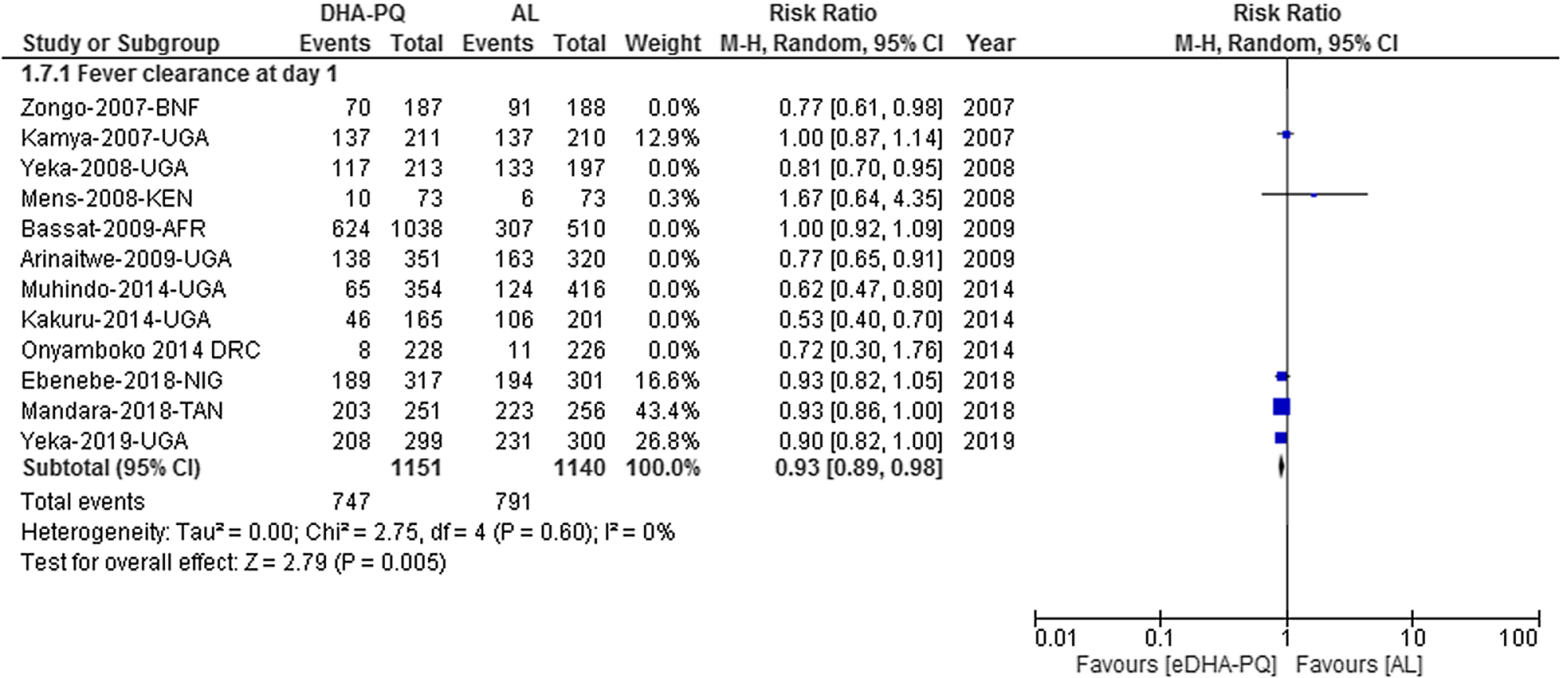
Forest plot of comparisonbetween dihydroartemisinin-piperaquine and artemether-lumefantrine for treatment of uncomplicated falciparum malaria among children in Africa on fever clearance on day 1

Fever clearance on day 1 was higher in patients who were treated with the DHA-PQ than AL [RR 0.93, 95% CI 0.89–0.98; participants = 2,291; studies = 12; I^2^ = 0% (Fig. 10)]. On day 2, fever had resolved in the majority of the patients regardless of the treatment group [RR 0.86, 95% CI 0.71–1.04; participants = 4971; studies = 11; I^2^ = 31% (Additional file 10)]. On day 3, fever had resolved in the majority of the patients regardless of the treatment group [RR 1.07, 95% CI 0.85–1.34; participants = 4664; studies = 11; I^2^ = 0% (Additional file 10)].

### Parasite clearance

**Fig. 11:**
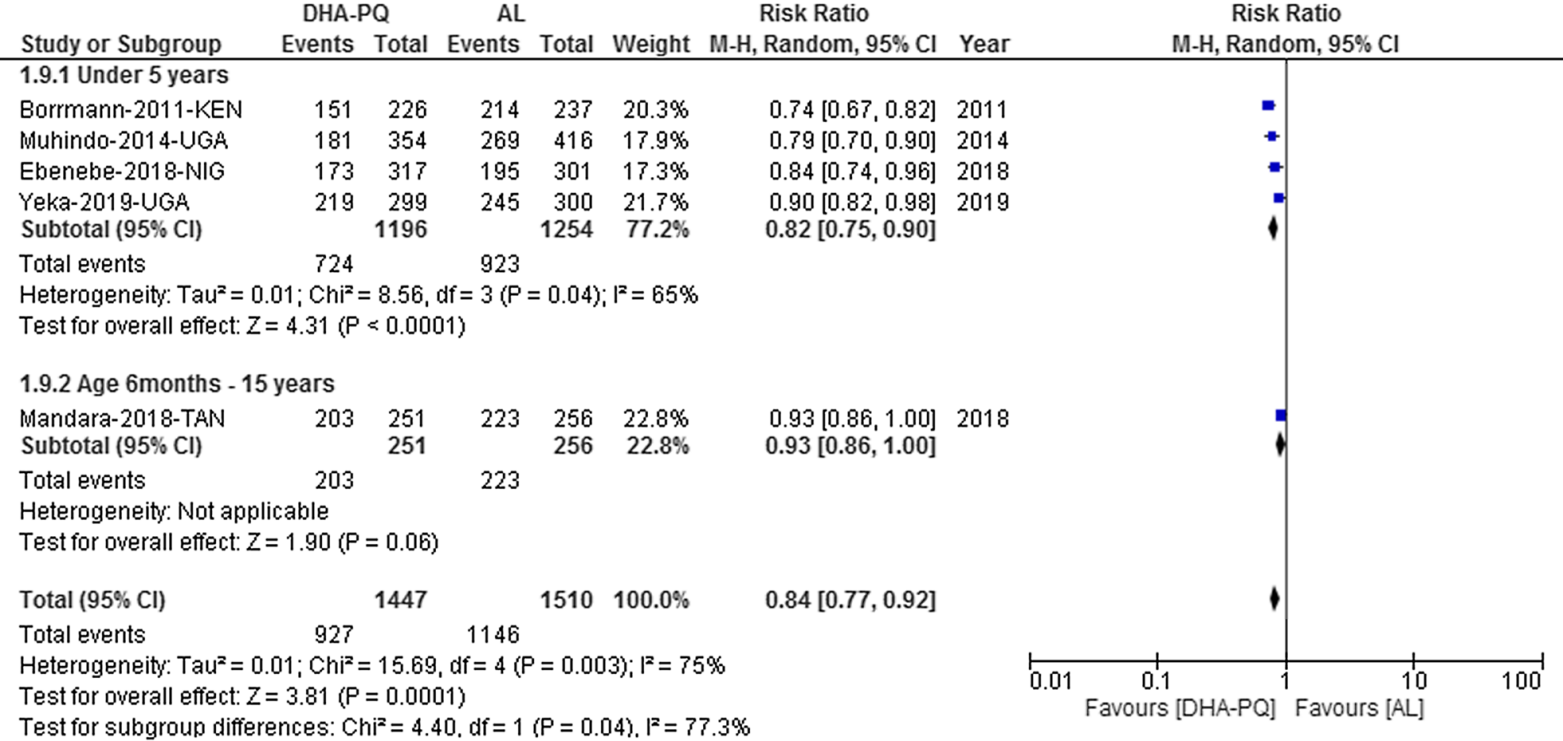
Forest plot of comparison between dihydroartemisinin-piperaquine and artemether-lumefantrine for treatment of uncomplicated falciparum malaria among children in Africa on parasite clearance on day 1

Parasite clearance at day 1 the pooled result had a considerable heterogeneity between studies [Tau^2^ = 0.01; Chi^2^ = 15.69, df = 4 (P = 0.003); I^2^ = 75% (Fig. 11)]. The result could not be pooled. On day 1, the percentage of patients with parasitaemia was significantly lower in the DHA-PQ treatment group than AL in under fives [RR 0.82, 95% CI 0.75–0.90; participants = 2450; studies = 4; I^2^ = 65% (Fig. 11)]. Similarly, the prevalence of parasitaemia in children between the age of 6 months to 15 years was significantly lower in DHA-PQ treatment group than AL [RR 0.93, 95% CI 0.86–1.00; participants = 507; studies = 1 (Fig. 11)].

#### Parasite clearance on day 2

The pooled result shows that the percentage of patients with parasitaemia on day 2 was significantly lower in patients who were treated with DHA-PQ than AL [RR 0.74, 95% CI 0.61–0.90; participants = 6065; studies = 13; I^2^ = 12% (Additional file 11)].

#### Parasite clearance at day 3

In the majority of studies the percentage of patients with detected parasitaemia was lower in both treatment groups on day 3 without significant difference [RR 0.99, 95% CI 0.50–1.98; participants = 6635; studies = 13; I^2^ = 0% (Additional file 11)].

### Quality of the evidence

The quality of the evidence in this review was assessed using the GRADE approach and presented the evidence in three summary of findings tables for efficacy [Summary of findings for the main comparison (Additional file 12)]. The evidence that DHA-PQ is more effective than that of AL on days 28 and 42 unadjusted by genotyping was of high and moderate quality of evidence. There was considerable heterogeneity between studies at days 28 and 42. In addition, DHA-PQ was consistently superior over AL on days 28 and 42 adjusted by genotyping was of high quality of evidence and both DHA-PQ and AL performed better than the WHO standard of 5% PCR-adjusted treatment failure on day 28 in all trials (high quality of evidence).

## Discussion

The therapeutic efficacy of anti-malarial drugs should be monitored regularly using the standard WHO protocol [51]. It involves assessing clinical and parasitological outcomes of treatment for at least 28 days post-treatment, and the appearance of the parasite in the blood is also monitored [2]. To distinguish true treatment failure from new infection, PCR genotyping should be used. If the PCR-corrected treatment failure is greater than the cut-off (≥ 10%), WHO recommends a change in the national malaria treatment policy [2, 51].

In this study, the 28 and 42 days PCR-uncorrected treatment failure of DHA-PQ was significantly lower than that of AL (high and moderate quality of evidence). There were 19/7,115 early treatment failures in the DHA-PQ group arm versus 30/6,083 in the AL arm. The 28 and 42-days PCR-corrected treatment failure in patients receiving DHA-PQ were significantly lower than those patients receiving AL (high quality of evidence). On day 28, the PCR-corrected treatment failure was below 5% in both treatment arms and a similar result was seen in the DHA-PQ treatment arm on day 42. On the contrary, a study from Burkina Faso [25] reported that the PCR-corrected treatment failure in the AL treatment arm was 28%. This result shows the need for national malaria treatment policy change because it was higher than the WHO cut-off (≥ 10%) [2]. However, the 63-days PCR-uncorrected and -corrected treatment failure in patients receiving DHA-PQ were not significantly different to those seen in patients receiving AL (moderate and high quality of evidence).

The observed lower PCR-unadjusted treatment failure on days 28 and 42 in the DHA-PQ treatment arm was similar with that of former reviews from Africa [5, 7]. As seen in Myanmar, Papua New Guinea, Angola, and elsewhere in Africa, recurrent parasitaemia due to recrudescence occurs significantly more frequently in those patients treated with AL in the first 28 days [21, 52–56]. In addition, DHA-PQ has shown extended post-treatment prophylactic effect in Africa, which decreased the risk of new infections after treatment compared to AL [7]. This difference might be attributed to the evening doses of AL given at home unsupervised, or to administration of AL without fatty food for less than 10% of lumefantrine is absorbed in empty stomach [57], and to the longer elimination half-life of piperaquine (23–28 days) compared with that for lumefantrine (3.2 days), which provides long-lasting post-treatment prophylactic effect [58, 59] and parasite dormancy and state of quiescence following ACT [60–62]. In addition, poor patient compliance and poor drug quality can play a major role in causing treatment failure [63]. For patients who live in areas where malaria transmission is higher and re-infection is likely, a longer post-treatment prophylactic period might have a great advantage [64], but due to the sub-therapeutic drug levels, selection for resistant parasites may occur [65]. For a patient who lives in the area where malaria transmission intensity is low, the benefit of the drug’s longer post-treatment prophylactic period is low and the probability of developing drug resistance is higher [66]. Using these drugs with longer post-treatment elimination half-life in these settings might be disadvantageous.

However, no significant difference was seen on PCR-adjusted treatment failure between the two treatment groups on days 28 and 42 [67, 68]. Similarly, a former review reported that in Papua New Guinea and Asia no significant difference between the two treatment groups was seen on days 28 and 63 [7]. During malaria epidemics, a drug with a longer prophylactic effect might limit presumptuous transmission of malaria. On the contrary, AL has shown a significant reduction in PCR-adjusted treatment failure in Asia and Senegal on days 28 and 42, respectively [5, 21, 69].

In this review, many children under the age of five who were treated with DHA-PQ had parasite clearance on day 1. Consistently, on day 2, participants from the DHA-PQ treatment group had faster parasite clearance than patients who were treated with AL. On day 3, the majority of the children in both treatment arms had parasite clearance. According to WHO [2, 22], suspected artemisinin resistance is defined as a delayed parasite clearance (slope half-life > 5 h or day 3 positivity rate > 10%). Although the predominant function of artemisinin is early parasite clearance, artemisinin component and partner drugs used in various ACT may also influence early parasite clearance. The absence of artemisinin resistance and the lower percentage of patients with detected parasitaemia regardless of the treatment group on day 3 observed in this study may suggest that *P. falciparum* remains sensitive to artemisinin derivatives. Other studies conducted in Papua New Guinea [52] and elsewhere in Africa and Papua New Guinea [70–72] reported that artemisinin resistance has not emerged in Africa and Papua New Guinea.

Some molecular studies in Africa show absence of the known *kelch-13 (k-13)* gene mutations associated with artemisinin resistance in Southeast Asia [19, 73, 74], implying that artemisinins are still effective and their capacity for parasite clearance has not changed. However, artemisinin resistance has been spreading in Southeast Asia [26, 75]; African countries have to be cautious about its potential emergence through continuous monitoring of parasite clearance and efficacy of ACT, and surveillance of polymorphism in the *k-13* gene.

In African settings, several risk factors were associated with persistent parasitaemia on days 1 and 2 after commencement of therapy [75]. Considering persistent parasitaemia on day 2, an elevated pre-treatment temperature, higher pre-treatment parasite density and being HIV infected were independently associated with a significantly increased risk of persistent parasitaemia [75].

The current malaria treatment guideline for *P. falciparum* is effective in Africa at present. However, insufficient drug levels, ineffectiveness of anti-malarial drugs, and drug resistance could lead to treatment failure. Further studies should be conducted in different African countries with different malaria transmission intensity to identify the risk factors for treatment failure.

### Study limitation

This study has two limitations. Most of the studies were not blinded and the efficacy assessments had potential for bias. This review could not provide strong evidence for the long-term, post- treatment prophylactic effect of the two drugs up to day 63.

## Conclusion

From this review, it can be concluded that DHA-PQ reduces new infection and recrudescence at days 28 and 42, more than AL. This may trigger DHA-PQ to become the first-line treatment option. Continuous studies to measure the efficacy of DHA-PQ and AL within 42 and 63 days follow-up are needed.

## Supplementary Information

**Additional file 1.** Detailed search strategy.

**Additional file 2.** Commands used for P-curve.

**Additional file 3.** Characteristics of excluded studies.

**Additional file 4.** Characteristics of included studies.

**Additional file 5.** Meta-regression of PCR-unadjusted treatment failure at day 28, association between age of the children and treatment failure.

**Additional file 6.** Meta-regression of PCR-unadjusted treatment failure at day 28, association between malaria transmission intensity within the countries and treatment failure.

**Additional file 7.** Funnel plot of comparison: dihydroartemisinin-piperaquine versus artemether-lumefantrine for treatment of uncomplicated falciparum malaria in African children, outcome: PCR-unadjusted treatment failure at day 28.

**Additional file 8.** Funnel plot of comparison: dihydroartemisinin-piperaquine versus artemether-lumefantrine for treatment of uncomplicated falciparum malaria in African children, outcome: PCR-adjusted treatment failure at day 28.

**Additional file 9.** Funnel plot of comparison: dihydroartemisinin-piperaquine versus artemether-lumefantrine for treatment of uncomplicated falciparum malaria in African children, outcome: PCR-adjusted treatment failure at day 42.

**Additional file 10.** Forest plot of comparison between dihydroartemisinin-piperaquine and artemether-lumefantrine for treatment of uncomplicated falciparum malaria in African children on fever clearance.

**Additional file 11.** Forest plot of comparison: dihydroartemisinin-piperaquine versus artemether-lumefantrine for treatment of uncomplicated falciparum malaria in African children, outcome: Parasite clearance day 2 and 3.

**Additional file 12**: Summary of finding tables.

## Abbreviations

ACT: Artemisinin-based combination therapy
ACPR: Adequate clinical and parasitological response
AL: Artemether-lumefantrine
BW: Body weight
CENTRAL: Cochrane Central Register of Controlled Trials
CI: Confidence interval
DHA-PQ: Dihydroartemisinin-piperaquine
ETF: Early treatment failure
GADE: Grading of recommendations, assessment, development, and evaluations
GOSH: Graphic display of heterogeneity
LCT: Late clinical failure
LPF: Late parasitological failure
PCR: Polymerase chain reaction
PICO: Population, intervention, comparison, and outcome
PRISMA: Preferred reporting items for systematic reviews and meta-analyses
RDT: Rapid diagnostic test
RR: Risk ratio
WHO: World Health Organization

## Declarations

### Availability of data and materials

All relevant data are within the manuscript and its supporting information files.
