# Supplementary material for: Efficacy of dihydroartemisinin-piperaquine versus artemether-lumefantrine for the treatment of uncomplicated Plasmodium falciparum malaria among children in Africa: a systematic review and meta-analysis of randomized control trials
Source: Malar J. 2021 Aug 12;20:340. doi: 10.1186/s12936-021-03873-1 (PMC8359548; doi:10.1186/s12936-021-03873-1)
Supplement: Supplementary file 1 — Additional file 1. Detailed search strategy. [file 12936_2021_3873_MOESM1_ESM.docx]

**Additional file S12: Summary of finding tables**

| **Treatment failure** | | | | | | |
| --- | --- | --- | --- | --- | --- | --- |
| **Dihydroartemisinin-piperaquine compared to artemether-lumefantrine for the treatment of uncomplicated *plasmodium falciparum* malaria among children in Africa.** | | | | | | |
| **Patient or population**: **African children with uncomplicated *plasmodium falciparum* malaria**  **Setting**: **Malaria endemic setting in Africa.**  **Intervention**: **Dihydroartemisinin-piperaquine**  **Comparison**: **Artemether-lumefantrine** | | | | | | |
| Outcomes | **Anticipated absolute effects^*^** (95% CI) | | Relative effect (95% CI) | № of participants  (studies) | Certainty of the evidence (GRADE) | Comments |
|  | **Risk with artemether-lumefantrine** | **Risk with dihydroartemisinin-piperaquine** |  |  |  |  |
| PCR unadjusted treatment failure at day 28 - Age 6months-15 years | 126 per 1,000 | **18 per 1,000** (10 to 33) | **RR 0.14** (0.08 to 0.26) | 1302 (4 RCTs) | ⨁⨁⨁⨁ HIGH ^a^ |  |
| PCR unadjusted treatment failure at day 28 - Under five years | 374 per 1,000 | **116 per 1,000** (86 to 157) | **RR 0.31** (0.23 to 0.42) | 14319 (13 RCTs) | ⨁⨁⨁◯ MODERATE ^a,b^ |  |
| PCR adjusted treatment failure at day 28 - PCR adjusted treatment failure at day 28 | 49 per 1,000 | **22 per 1,000** (14 to 33) | **RR 0.45** (0.29 to 0.68) | 8508 (16 RCTs) | ⨁⨁⨁⨁ HIGH ^a^ |  |
| PCR unadjusted treatment failure at day 42 - PCR unadjusted treatment failure at day 42 | 326 per 1,000 | **183 per 1,000** (156 to 215) | **RR 0.56** (0.48 to 0.66) | 7667 (17 RCTs) | ⨁⨁◯◯ LOW ^a,c,d^ |  |
| PCR adjusted treatment failure at day 42 - PCR adjusted treatment failure at day 42 | 55 per 1,000 | **33 per 1,000** (26 to 43) | **RR 0.60** (0.47 to 0.78) | 5959 (17 RCTs) | ⨁⨁⨁⨁ HIGH ^a^ |  |
| PCR unadjusted treatment failure at day 63 - PCR unadjusted treatment failure at day 63 | 440 per 1,000 | **269 per 1,000** (150 to 484) | **RR 0.61** (0.34 to 1.10) | 3365 (3 RCTs) | ⨁⨁⨁◯ MODERATE ^a,b,e^ |  |
| PCR adjusted treatment failure at day 63 - PCR adjusted treatment failure at day 63 | 51 per 1,000 | **44 per 1,000** (29 to 68) | **RR 0.87** (0.57 to 1.34) | 3384 (4 RCTs) | ⨁⨁⨁⨁ HIGH ^a,f^ |  |
| ***The risk in the intervention group** (and its 95% confidence interval) is based on the assumed risk in the comparison group and the **relative effect** of the intervention (and its 95% CI).  **CI:** Confidence interval; **RR:** Risk ratio | | | | | | |
| **GRADE Working Group grades of evidence** **High certainty:** We are very confident that the true effect lies close to that of the estimate of the effect **Moderate certainty:** We are moderately confident in the effect estimate: The true effect is likely to be close to the estimate of the effect, but there is a possibility that it is substantially different **Low certainty:** Our confidence in the effect estimate is limited: The true effect may be substantially different from the estimate of the effect **Very low certainty:** We have very little confidence in the effect estimate: The true effect is likely to be substantially different from the estimate of effect | | | | | | |
