# Supplementary material for: Efficacy of dihydroartemisinin-piperaquine versus artemether-lumefantrine for the treatment of uncomplicated Plasmodium falciparum malaria among children in Africa: a systematic review and meta-analysis of randomized control trials
Source: Malar J. 2021 Aug 12;20:340. doi: 10.1186/s12936-021-03873-1 (PMC8359548; doi:10.1186/s12936-021-03873-1)
Supplement: Supplementary file 2 — Additional file 2. Commands used for P-curve. [file 12936_2021_3873_MOESM2_ESM.docx]

Additional file S1: Detailed search strategy

| **Search set** | **CENTRAL ^a^** | **MEDLINE** | **EMBASE** |
| --- | --- | --- | --- |
|  | malaria | malaria | malaria |
|  | arte* | arte* | arte* |
|  | dihydroarte* | dihydroarte* | dihydroarte* |
|  | lumefantrine | lumefantrine | lumefantrine |
|  | Coartem* | Coartem* | Coartem* |
|  | 2 or 3 | 2 or 3 | 2 or 3 |
|  | 4 or 5 | 4 or 5 | 4 or 5 |
|  | 1 and 6 and 7 | 1 and 6 and 7 | 1 and 6 and 7 |
|  | Limit 8 to date between 2004-2021 | Limit 8 to humans | Limit 8 to humans |
|  |  | Limit 8 to randomized control trial | Limit 8 to randomized control trial |
|  |  | Limit 8 to date between 2004-2021 | Limit 8 to date between 2004-2021 |

^a^Cochrane Infectious Diseases Group Specialized Register.

^b^Search terms used in combination with the search strategy for retrieving trials developed by The Cochrane Collabortion (Lefebvre C 2021 [142] ) upper case: MeSH or EMTREE heading; lower case: free text term.
