# Supplementary material for: Efficacy of dihydroartemisinin-piperaquine versus artemether-lumefantrine for the treatment of uncomplicated Plasmodium falciparum malaria among children in Africa: a systematic review and meta-analysis of randomized control trials
Source: Malar J. 2021 Aug 12;20:340. doi: 10.1186/s12936-021-03873-1 (PMC8359548; doi:10.1186/s12936-021-03873-1)
Supplement: Supplementary file 3 — Additional file 3. Characteristics of excluded studies. [file 12936_2021_3873_MOESM3_ESM.docx]

Additional file S 2: Commands used for P-curve

#' Perform a \emph{p}-curve analysis

#'

#' This function performs a \eqn{p}-curve analysis using a \code{meta} object or calculated effect size data.

#'

#' @usage pcurve(x, effect.estimation = FALSE, N, dmin = 0, dmax = 1)

#'

#' @param x Either an object of class \code{meta}, generated by the \code{metagen}, \code{metacont},

#' \code{metacor}, \code{metainc}, or \code{metabin} function, or a dataframe containing the calculated effect size

#' (named \code{TE}, log-transformed if based on a ratio), standard error (named \code{seTE}) and study label (named \code{studlab})

#' for each study.

#' @param effect.estimation Logical. Should the true effect size underlying the \emph{p}-curve be estimated?

#' If set to \code{TRUE}, a vector containing the total sample size for each study must be provided for

#' \code{N}. \code{FALSE} by default.

#' @param N A numeric vector of same length as the number of effect sizes included in \code{x} specifying the

#' total sample size \eqn{N} corresponding to each effect. Only needed if \code{effect.estimation = TRUE}.

#' @param dmin If \code{effect.estimation = TRUE}: lower limit for the effect size (\eqn{d}) space in which

#' the true effect size should be searched. Must be greater or equal to 0. Default is 0.

#' @param dmax If \code{effect.estimation = TRUE}: upper limit for the effect size (\eqn{d}) space in which

#' the true effect size should be searched. Must be greater than 0. Default is 1.

#'

#' @details

#' \strong{P-curve Analysis}

#'

#' \eqn{P}-curve analysis (Simonsohn, Simmons & Nelson, 2014, 2015) has been proposed as a method

#' to detect \eqn{p}-hacking and publication bias in meta-analyses.

#'

#' \eqn{P}-Curve assumes that publication bias

#' is not only generated because researchers do not publish non-significant results,

#' but also because analysts “play” around with their data ("\eqn{p}-hacking"; e.g., selectively removing outliers,

#' choosing different outcomes, controlling for different variables) until a non-significant

#' finding becomes significant (i.e., \eqn{p<0.05}).

#'

#' The method assumes that for a specific research

#' question, \eqn{p}-values smaller 0.05 of included studies should follow a right-skewed distribution

#' if a true effect exists, even when the power in single studies was (relatively) low. Conversely,

#' a left-skewed \eqn{p}-value distribution indicates the presence of \eqn{p}-hacking and absence of

#' a true underlying effect. To control for "ambitious" \eqn{p}-hacking, \eqn{P}-curve also incorporates a

#' "half-curve" test (Simonsohn, Simmons & Nelson, 2014, 2015).

#'

#' Simonsohn et al. (2014)

#' stress that \eqn{p}-curve analysis should only be used for test statistics which were actually of interest

#' in the context of the included study, and that a detailed table documenting the reported results

#' used in for the \eqn{p}-curve analysis should be created before communicating

#' results (\href{http://www.p-curve.com/Supplement/}{link}).

#'

#' \strong{Implementation in the function}

#'

#' To generate the \eqn{p}-curve and conduct the analysis, this function reuses parts of the \emph{R} code underlying

#' the \href{http://p-curve.com/app4/pcurve_app4.052.r}{P-curve App 4.052} (Simonsohn, 2017). The effect sizes

#' included in the \code{meta} object or \code{data.frame} provided for \code{x} are transformed

#' into \eqn{z}-values internally, which are then used to calculate {p}-values and conduct the

#' Stouffer and Binomial test used for the \eqn{p}-curve analysis. Interpretations of the function

#' concerning the presence or absence/inadequateness of evidential value are made according to the

#' guidelines described by Simonsohn, Simmons and Nelson (2015):

#'

#' \itemize{

#' \item \strong{Evidential value present}: The right-skewness test is significant for the half curve with

#' \eqn{p<0.05} \strong{or} the \eqn{p}-value of the right-skewness test is \eqn{<0.1} for both the half and full curve.

#' \item \strong{Evidential value absent or inadequate}: The flatness test is \eqn{p<0.05} for the full curve

#' \strong{or} the flatness test for the half curve and the binomial test are \eqn{p<0.1}.

#'}

#'

#' For effect size estimation, the \code{pcurve} function implements parts of the loss function

#' presented in Simonsohn, Simmons and Nelson (2014b).

#' The function generates a loss function for candidate effect sizes \eqn{\hat{d}}, using \eqn{D}-values in

#' a Kolmogorov-Smirnov test as the metric of fit, and the value of \eqn{\hat{d}} which minimizes \eqn{D}

#' as the estimated true effect.

#'

#' It is of note that a lack of robustness of \eqn{p}-curve analysis results

#' has been noted for meta-analyses with substantial heterogeneity (van Aert, Wicherts, & van Assen, 2016).

#' Following van Aert et al., adjusted effect size estimates should only be

#' reported and interpreted for analyses with \eqn{I^2} values below 50 percent.

#' A warning message is therefore printed by

#' the \code{pcurve} function when \code{x} is of class \code{meta} and the between-study heterogeneity

#' of the meta-analysis is substantial (i.e., \eqn{I^2} greater than 50 percent).

#'

#'

#' @references Harrer, M., Cuijpers, P., Furukawa, T.A, & Ebert, D. D. (2019).

#' \emph{Doing Meta-Analysis in R: A Hands-on Guide}. DOI: 10.5281/zenodo.2551803.

#' \href{https://bookdown.org/MathiasHarrer/Doing_Meta_Analysis_in_R/pcurve.html}{Chapter 9.2}.

#'

#' Simonsohn, U., Nelson, L. D., & Simmons, J. P. (2014a). P-curve: a Key to the File-drawer.

#' \emph{Journal of Experimental Psychology, 143}(2), 534.

#'

#' Simonsohn, U., Nelson, L. D. & Simmons, J. P. (2014b). P-Curve and Effect Size:

#' Correcting for Publication Bias Using Only Significant Results.

#' \emph{Perspectives on Psychological Science 9}(6), 666–81.

#'

#' Simonsohn, U., Nelson, L. D. & Simmons, J. P. (2015). Better P-Curves: Making P-Curve

#' Analysis More Robust to Errors, Fraud, and Ambitious P-Hacking, a Reply to Ulrich and Miller (2015).

#' \emph{Journal of Experimental Psychology, 144}(6), 1146-1152.

#'

#' Simonsohn, U. (2017). R code for the P-Curve App 4.052. http://p-curve.com/app4/pcurve_app4.052.r (Accessed 2019-08-16).

#'

#' Van Aert, R. C., Wicherts, J. M., & van Assen, M. A. (2016).

#' Conducting meta-analyses based on p values: Reservations and recommendations for applying

#' \emph{p}-uniform and \emph{p}-curve. \emph{Perspectives on Psychological Science, 11}(5), 713-729.

#'

#' @author Mathias Harrer & David Daniel Ebert

#'

#' @return Returns a plot and main results of the pcurve analysis:

#' \itemize{

#' \item \strong{P-curve plot}: A plot displaying the observed \eqn{p}-curve and significance results

#' for the right-skewness and flatness test.

#' \item \strong{Number of studies}: The number of studies provided for the analysis, the number

#' of significant \eqn{p}-values included in the analysis, and the number of studies with \eqn{p<0.025}

#' used for the half-curve tests.

#' \item \strong{Test results}: The results for the right-skewness and flatness test, including the

#' \eqn{p_{binomial}} value, as well as the \eqn{z} and \eqn{p} value for the full and half-curve test.

#' \item \strong{Power Estimate}: The power estimate and 95\% confidence interval.

#' \item \strong{Evidential value}: Two lines displaying if evidential value is present and/or absent/inadequate based

#' on the results (using the guidelines by Simonsohn et al., 2015, see Details).

#' \item \strong{True effect estimate}: If \code{effect.estimation} is set to \code{TRUE}, the estimated true effect

#' \eqn{\hat{d}} is returned additionally.

#'}

#'

#'

#' If results are saved to a variable, a list of class \code{pcurve} containing the following objects is returned:

#' \itemize{

#' \item \code{pcurveResults}: A data frame containing the results for the right-skewness and flatness test, including the

#' \eqn{p_{binomial}} value, as well as the \eqn{z} and \eqn{p} value for the full and half-curve test.

#' \item \code{Power}: The power estimate and 95\% confidence interval.

#' \item \code{PlotData}: A data frame with the data used in the \eqn{p}-curve plot.

#' \item \code{Input}: A data frame containing the provided effect sizes, calculated \eqn{p}-values and individual results for each included (significant) effect.

#' \item \code{EvidencePresent}, \code{EvidenceAbsent}, \code{kInput}, \code{kAnalyzed}, \code{kp0.25}: Further results of the \eqn{p}-curve analysis, including the presence/absence of evidence interpretation,

#' and number of provided/significant/\eqn{p<0.025} studies.

#' \item \code{I2}: \eqn{I^2}-Heterogeneity of the studies provided as input (only when \code{x} is of class \code{meta}).

#' \item \code{class.meta.object}: \code{class} of the original object provided in \code{x}.

#'}

#'

#'

#' @import stringr poibin

#' @importFrom graphics abline axis lines mtext par plot points rect segments text

#' @importFrom stats as.formula hat influence ks.test optimize pbinom pchisq pf pnorm pt punif qchisq qf qnorm qt reformulate reorder setNames uniroot

#'

#' @export pcurve

#'

#' @seealso

#' \code{\link{eggers.test}}

#'

#' @examples

#' # Example 1: Use metagen object, do not estimate d

#' suppressPackageStartupMessages(library(meta))

#' data("ThirdWave")

#' meta1 = metagen(TE,seTE, studlab=ThirdWave$Author, data=ThirdWave)

#' pcurve(meta1)

#'

#' # Example 2: Provide Ns, calculate d estimate

#' N = c(105, 161, 60, 37, 141, 82, 97, 61, 200, 79, 124, 25, 166, 59, 201, 95, 166, 144)

#' pcurve(meta1, effect.estimation = TRUE, N = N)

#'

#' # Example 3: Use metacont object, calculate d estimate

#' data("amlodipine")

#' meta2 <- metacont(n.amlo, mean.amlo, sqrt(var.amlo),

#' n.plac, mean.plac, sqrt(var.plac),

#' data=amlodipine, studlab=study, sm="SMD")

#' N = amlodipine$n.amlo + amlodipine$n.plac

#' pcurve(meta2, effect.estimation = TRUE, N = N, dmin = 0, dmax = 1)

#'

#' # Example 4: Construct x object from scratch

#' sim = data.frame("studlab" = c(paste("Study_", 1:18, sep = "")),

#' "TE" = c(0.561, 0.296, 0.648, 0.362, 0.770, 0.214, 0.476,

#' 0.459, 0.343, 0.804, 0.357, 0.476, 0.638, 0.396, 0.497,

#' 0.384, 0.568, 0.415),

#' "seTE" = c(0.338, 0.297, 0.264, 0.258, 0.279, 0.347, 0.271, 0.319,

#' 0.232, 0.237, 0.385, 0.398, 0.342, 0.351, 0.296, 0.325,

#' 0.322, 0.225))

#' pcurve(sim)

pcurve = function(x, effect.estimation = FALSE, N, dmin = 0, dmax = 1){

# Rename x to metaobject, remove x

metaobject = x

rm(x)

# Stop if metaobject is not meta or does not contain TE or seTE column

if (!(class(metaobject)[1] %in% c("metagen", "metabin", "metacont", "metacor", "metainc", "meta", "metaprop"))){

for (i in 1:length(colnames(metaobject))){

te.exists = FALSE

if (colnames(metaobject)[i]=="TE"){

te.exists = TRUE

break

} else {

}

}

for (i in 1:length(colnames(metaobject))){

sete.exists = FALSE

if (colnames(metaobject)[i]=="seTE"){

sete.exists = TRUE

break

} else {

}

}

for (i in 1:length(colnames(metaobject))){

studlab.exists = FALSE

if (colnames(metaobject)[i]=="studlab"){

studlab.exists = TRUE

break

} else {

}

}

if(te.exists == FALSE | sete.exists ==FALSE | studlab.exists ==FALSE){

stop("x must be a meta-analysis object generated by meta functions or a data.frame with columns labeled studlab, TE, and seTE.")

}

}

#Disable scientific notation

options(scipen=999)

# Calculate Z

zvalues.input = abs(metaobject$TE/metaobject$seTE)

##############################################

# 1. Functions ###############################

##############################################

getncp.f =function(df1,df2, power) {

error = function(ncp_est, power, x, df1,df2) pf(x, df1 = df1, df2=df2, ncp = ncp_est) - (1-power)

xc=qf(p=.95, df1=df1,df2=df2)

return(uniroot(error, c(0, 1000), x = xc, df1 = df1,df2=df2, power=power)$root) }

getncp.c =function(df, power) {

xc=qchisq(p=.95, df=df)

error = function(ncp_est, power, x, df) pchisq(x, df = df, ncp = ncp_est) - (1-power)

return(uniroot(error, c(0, 1000), x = xc, df = df, power=power)$root) }

getncp=function(family,df1,df2,power) {

if (family=="f") ncp=getncp.f(df1=df1,df2=df2,power=power)

if (family=="c") ncp=getncp.c(df=df1,power=power)

return(ncp) }

percent <- function(x, digits = 0, format = "f", ...) {

paste(formatC(100 * x, format = format, digits = digits, ...), "%", sep = "")

}

pbound=function(p) pmin(pmax(p,2.2e-16),1-2.2e-16)

prop33=function(pc)

{

prop=ifelse(family=="f" & p<.05,1-pf(qf(1-pc,df1=df1, df2=df2),df1=df1, df2=df2, ncp=ncp33),NA)

prop=ifelse(family=="c" & p<.05,1-pchisq(qchisq(1-pc,df=df1), df=df1, ncp=ncp33),prop)

prop

}

stouffer=function(pp) sum(qnorm(pp),na.rm=TRUE)/sqrt(sum(!is.na(pp)))

###############################################################################

# 2. Process data ############################################################

###############################################################################

# Note: due to reliance on the pcurve-app function, z-scores are pasted into characters first

# and then screened to generate variables necessary for further computation

zvalues.input = paste("z=", zvalues.input, sep="")

filek = "input"

raw = zvalues.input

raw=tolower(raw)

ktot=length(raw)

k=seq(from=1,to=length(raw))

stat=substring(raw,1,1)

test=ifelse(stat=="r","t",stat)

# Create family

family=test

family=ifelse(test=="t","f",family)

family=ifelse(test=="z","c",family)

#family: f,c converting t-->f and z-->c

# Find comma,parentheses,equal sign

par1 =str_locate(raw,"\\(")[,1]

par2 =str_locate(raw,"\\)")[,1]

comma=str_locate(raw,",")[,1]

eq =str_locate(raw,"=")[,1]

# DF for t-tests

df=as.numeric(ifelse(test=="t",substring(raw,par1+1,par2 -1),NA))

# DF1

df1=as.numeric(ifelse(test=="f",substring(raw,par1+1,comma-1),NA))

df1=as.numeric(ifelse(test=="z",1,df1))

df1=as.numeric(ifelse(test=="t",1,df1))

df1=as.numeric(ifelse(test=="c",substring(raw,par1+1,par2 -1),df1))

# DF2

df2=as.numeric(ifelse(test=="f",substring(raw,comma+1,par2-1),NA))

df2=as.numeric(ifelse(test=="t",df,df2))

equal=abs(as.numeric(substring(raw,eq+1)))

value=ifelse((stat=="f" | stat=="c"),equal,NA)

value=ifelse(stat=="r", (equal/(sqrt((1-equal**2)/df2)))**2,value)

value=ifelse(stat=="t", equal**2 ,value)

value=ifelse(stat=="z", equal**2 ,value)

p=ifelse(family=="f",1-pf(value,df1=df1,df2=df2),NA)

p=ifelse(family=="c",1-pchisq(value,df=df1),p)

p=pbound(p) #Bound it to level of precision, see function 3 above

ksig= sum(p<.05,na.rm=TRUE) #significant studies

khalf=sum(p<.025,na.rm=TRUE) #half p-curve studies

if (ksig <= 2){

stop("Two or less significant (p<0.05) effect sizes were detected, so p-curve analysis cannot be conducted.")

}

##############################################################################

# 3. PP-values ###############################################################

##############################################################################

# Right Skew, Full p-curve

ppr=as.numeric(ifelse(p<.05,20*p,NA))

ppr=pbound(ppr)

# Right Skew, half p-curve

ppr.half=as.numeric(ifelse(p<.025,40*p,NA))

ppr.half=pbound(ppr.half)

# Power of 33%

ncp33=mapply(getncp,df1=df1,df2=df2,power=1/3,family=family)

# Full-p-curve

pp33=ifelse(family=="f" & p<.05,3*(pf(value, df1=df1, df2=df2, ncp=ncp33)-2/3),NA)

pp33=ifelse(family=="c" & p<.05,3*(pchisq(value, df=df1, ncp=ncp33)-2/3),pp33)

pp33=pbound(pp33)

# half p-curve

prop25=3*prop33(.025)

prop25.sig=prop25[p<.05]

#Compute pp-values for the half

pp33.half=ifelse(family=="f" & p<.025, (1/prop25)*(pf(value,df1=df1,df2=df2,ncp=ncp33)-(1-prop25)),NA)

pp33.half=ifelse(family=="c" & p<.025, (1/prop25)*(pchisq(value,df=df1, ncp=ncp33)-(1-prop25)),pp33.half)

pp33.half=pbound(pp33.half)

##############################################################################

# 4. Stouffer & Binomial test ################################################

##############################################################################

# Convert pp-values to Z scores, using Stouffer function above

Zppr = stouffer(ppr)

Zpp33 = stouffer(pp33)

Zppr.half = stouffer(ppr.half)

Zpp33.half = stouffer(pp33.half)

# Overall p-values from Stouffer test

p.Zppr = pnorm(Zppr)

p.Zpp33 = pnorm(Zpp33)

p.Zppr.half = pnorm(Zppr.half)

p.Zpp33.half = pnorm(Zpp33.half)

# Save results to file

main.results=as.numeric(c(ktot, ksig, khalf, Zppr,

p.Zppr, Zpp33, p.Zpp33, Zppr.half,

p.Zppr.half, Zpp33.half, p.Zpp33.half))

# BINOMIAL

# Observed share of p<.025

prop25.obs=sum(p<.025)/sum(p<.05)

# Flat null

binom.r=1-pbinom(q=prop25.obs*ksig- 1, prob=.5, size=ksig)

# Power of 33% null

binom.33=ppoibin(kk=prop25.obs*ksig,pp=prop25[p<.05])

# Save binomial results

binomial=c(mean(prop25.sig), prop25.obs, binom.r, binom.33)

# Beautifyier Function

cleanp=function(p)

{

p.clean=round(p,4) #Round it

p.clean=substr(p.clean,2,6) #Drop the 0

p.clean=paste0("= ",p.clean)

if (p < .0001) p.clean= " < .0001"

if (p > .9999) p.clean= " > .9999"

return(p.clean)

}

#If there are zero p<.025, change Stouffer values for half-p-curve tests for "N/A" messages

if (khalf==0) {

Zppr.half ="N/A"

p.Zppr.half ="=N/A"

Zpp33.half ="N/A"

p.Zpp33.half ="=N/A"

}

#If there are more than 1 p<.025, round the Z and beutify the p-values

if (khalf>0) {

Zppr.half =round(Zppr.half,2)

Zpp33.half =round(Zpp33.half,2)

p.Zppr.half=cleanp(p.Zppr.half)

p.Zpp33.half=cleanp(p.Zpp33.half)

}

#Clean results for full test

Zppr=round(Zppr,2)

Zpp33=round(Zpp33,2)

p.Zppr=cleanp(p.Zppr)

p.Zpp33=cleanp(p.Zpp33)

binom.r=cleanp(binom.r)

binom.33=cleanp(binom.33)

################################################

# 5. Power ####################################

################################################

powerfit=function(power_est)

{

ncp_est=mapply(getncp,df1=df1,df2=df2,power=power_est,family=family)

pp_est=ifelse(family=="f" & p<.05,(pf(value,df1=df1,df2=df2,ncp=ncp_est)-(1-power_est))/power_est,NA)

pp_est=ifelse(family=="c" & p<.05,(pchisq(value,df=df1,ncp=ncp_est)-(1-power_est))/power_est,pp_est)

pp_est=pbound(pp_est)

return(stouffer(pp_est))

}

fit=c()

fit=abs(powerfit(.051))

for (i in 6:99) fit=c(fit,abs(powerfit(i/100)))

mini=match(min(fit,na.rm=TRUE),fit)

hat=(mini+4)/100

x.power=seq(from=5,to=99)/100

get.power_pct =function(pct) {

#Function that finds power that gives p-value=pct for the Stouffer test

#for example, get.power_pct(.5) returns the level of power that leads to p=.5 for the stouffer test.

#half the time we would see p-curves more right skewed than the one we see, and half the time

#less right-skewed, if the true power were that get.power_pct(.5). So it is the median estimate of power

#similarliy, get.power_pct(.1) gives the 10th percentile estimate of power...

#Obtain the normalized equivalent of pct, e.g., for 5% it is -1.64, for 95% it is 1.64

z=qnorm(pct) #convert to z because powerfit() outputs a z-score.

#Quantify gap between computed p-value and desired pct

error = function(power_est, z) powerfit(power_est) - z

#Find the value of power that makes that gap zero, (root)

return(uniroot(error, c(.0501, .99),z)$root) }

# Boundary conditions

p.power.05=pnorm(powerfit(.051)) #Proability p-curve would be at least at right-skewed if power=.051

p.power.99=pnorm(powerfit(.99)) #Proability p-curve would be at least at right-skewed if power=.99

# Lower end of ci

if (p.power.05<=.95) power.ci.lb=.05

if (p.power.99>=.95) power.ci.lb=.99

if (p.power.05>.95 && p.power.99<.95) power.ci.lb=get.power_pct(.95)

# Higher end of CI

if (p.power.05<=.05) power.ci.ub=.05

if (p.power.99>=.05) power.ci.ub=.99

if (p.power.05>.05 && p.power.99<.05) power.ci.ub=get.power_pct(.05)

# Save power fit

power_results=c(power.ci.lb,hat,power.ci.ub)

##############################################################################

# 6. Plot ###################################################################

##############################################################################

# Green line (Expected p-curve for 33% power)

gcdf1=prop33(.01)

gcdf2=prop33(.02)

gcdf3=prop33(.03)

gcdf4=prop33(.04)

green1=mean(gcdf1,na.rm=TRUE)*3

green2=mean(gcdf2-gcdf1,na.rm=TRUE)*3

green3=mean(gcdf3-gcdf2,na.rm=TRUE)*3

green4=mean(gcdf4-gcdf3,na.rm=TRUE)*3

green5=mean(1/3-gcdf4,na.rm=TRUE)*3

green=100*c(green1,green2,green3,green4,green5)

# Blue line (observed p-curve)

ps=ceiling(p[p<.05]*100)/100

blue=c()

for (i in c(.01,.02,.03,.04,.05)) blue=c(blue,sum(ps==i,na.rm=TRUE)/ksig*100)

# Red line

red=c(20,20,20,20,20)

# Make the graph

x = c(.01,.02,.03,.04,.05)

par(mar=c(6,5.5,1.5,3))

moveup=max(max(blue[2:5])-66,0)

ylim=c(0,105+moveup)

legend.top=100+moveup

plot(x,blue, type='l', col='dodgerblue2', main="",

lwd=2, xlab="", ylab="", xaxt="n",yaxt="n", xlim=c(0.01,0.051),

ylim=ylim, bty='L', las=1,axes=F)

x_=c(".01",".02",".03",".04",".05")

axis(1,at=x,labels=x_)

y_=c("0%","25%","50%","75%","100%")

y=c(0,25,50,75,100)

axis(2,at=y,labels=y_,las=1,cex.axis=1.2)

mtext("Percentage of test results",font=2,side=2,line=3.85,cex=1.25)

mtext("p ",font=4,side=1,line=2.3,cex=1.25)

mtext(" -value", font=2,side=1,line=2.3,cex=1.25)

points(x,blue,type="p",pch=20,bg="dodgerblue2",col="dodgerblue2")

text(x+.00075,blue+3.5,percent(round(blue)/100),col='black', cex=.75)

lines(x,red, type='l', col='firebrick2', lwd=1.5, lty=3)

lines(x,green, type='l', col='springgreen4', lwd=1.5, lty=5)

tab1=.017 #Labels for line at p=.023 in x-axis

tab2=tab1+.0015 #Test results and power esimates at tab1+.0015

gap1=9 #between labels

gap2=4 #between lable and respective test (e.g., "OBserved p-curve" and "power estimate")

font.col='gray44'

text.blue=paste0("Power estimate: ",percent(hat),", CI(",

percent(power.ci.lb),",",

percent(power.ci.ub),")")

text(tab1,legend.top, adj=0,cex=.85,bquote("Observed "*italic(p)*"-curve"))

text(tab2,legend.top-gap2,adj=0,cex=.68,text.blue,col=font.col)

text.red=bquote("Tests for right-skewness: "*italic(p)*""[Full]~.(p.Zppr)*", "*italic(p)*""[Half]~.(p.Zppr.half))

#note: .() within bquote prints the value rather than the variable name

text(tab1,legend.top-gap1, adj=0,cex=.85, "Null of no effect" )

text(tab2,legend.top-gap1-gap2, adj=0,cex=.68, text.red, col=font.col )

text.green=bquote("Tests for flatness: "*italic(p)*""[Full]~.(p.Zpp33)*", "*italic(p)*""[half]~.(p.Zpp33.half)*", "*italic(p)*""[Binomial]~.(binom.33))

text(tab1,legend.top-2*gap1, adj=0,cex=.85,"Null of 33% power")

text(tab2,legend.top-2*gap1-gap2, adj=0,cex=.68,text.green,col=font.col)

segments(x0=tab1-.005,x1=tab1-.001,y0=legend.top,y1=legend.top, col='dodgerblue2',lty=1,lwd=1.5)

segments(x0=tab1-.005,x1=tab1-.001,y0=legend.top-gap1, y1=legend.top-gap1,col='firebrick2',lty=3,lwd=1.5)

segments(x0=tab1-.005,x1=tab1-.001,y0=legend.top-2*gap1,y1=legend.top-2*gap1,col='springgreen4',lty=2,lwd=1.5)

rect(tab1-.0065,legend.top-2*gap1-gap2-3,tab1+.032,legend.top+3,border='gray85')

msgx=bquote("Note: The observed "*italic(p)*"-curve includes "*.(ksig)*

" statistically significant ("*italic(p)*" < .05) results, of which "*.(khalf)*

" are "*italic(p)*" < .025.")

mtext(msgx,side=1,line=4,cex=.65,adj=0)

kns=ktot-ksig

if (kns==0) ns_msg="There were no non-significant results entered."

if (kns==1) ns_msg=bquote("There was one additional result entered but excluded from "*italic(p)*"-curve because it was "*italic(p)*" > .05.")

if (kns>1) ns_msg=bquote("There were "*.(kns)*" additional results entered but excluded from "*italic(p)*"-curve because they were "*italic(p)*" > .05.")

mtext(ns_msg,side=1,line=4.75,cex=.65,adj=0)

##############################################################################

# 7 Save Calculations #######################################################

##############################################################################

# table_calc

table_calc=data.frame(raw, p, ppr, ppr.half, pp33, pp33.half,

qnorm(ppr), qnorm(ppr.half), qnorm(pp33), qnorm(pp33.half))

headers1=c("Entered statistic","p-value", "ppr", "ppr half", "pp33%","pp33 half",

"Z-R","Z-R half","Z-33","z-33 half")

table_calc=setNames(table_calc,headers1)

# table_figure

headers2=c("p-value","Observed (blue)","Power 33% (Green)", "Flat (Red)")

table_figure=setNames(data.frame(x,blue,green,red),headers2)

################################################

# 8. Cumulative p-curves (Deprecated) ##########

################################################

#7.1 FUNCTION THAT RECOMPUTES OVERALL STOUFFER TEST WITHOUT (K) MOST EXTREME VALUES, ADJUSTING THE UNIFORM TO ONLY INCLUDE RANGE THAT REMAINS

dropk=function(pp,k,droplow)

{

#Syntax:

#pp: set of pp-values to analyze sensitivity to most extremes

#k: # of most extreme values to exclude

#dropsmall: 1 to drop smallest, 0 to drop largest

pp=pp[!is.na(pp)] #Drop missing values

n=length(pp) #See how many studies are left

pp=sort(pp) #Sort the pp-value from small to large

if (k==0) ppk=pp #If k=0 do nothing for nothing is being dropped

#If we are dropping low values

if (droplow==1 & k>0)

{

#Eliminate lowest k from the vector of pp-values

ppk=(pp[(1+k):n])

ppmin=min(pp[k],k/(n+1)) #Boundary used to define possible range of values after exclusion

ppk=(ppk-ppmin)/(1-ppmin) #Take the k+1 smallest pp-value up to the highest, subtract from each the boundary value, divide by the range, ~U(0,1) under the null

#This is explained in Supplement 1 of Simonsohn, Simmons Nelson, JEPG 2016 "Better p-curves" paper. See https://osf.io/mbw5g/

}

#If we are dropping high values

if (droplow==0 & k>0)

{

#Eliminate lowest k from the vector of pp-values

ppk=pp[1:(n-k)]

ppmax=max(pp[n-k+1],(n-k)/(n+1)) #Find new boundary of range

ppk=ppk/ppmax #Redefine range to make U(0,1)

}

#In case of a tie with two identical values we would have the ppk be 0 or 1, let's replace that with almost 0 and almost 1

ppk=pmax(ppk,.00001) #Adds small constant to the smallest redefined p-value, avoids problem if dropped p-value is "equal" to next highest, then that pp-value becomes 0

ppk=pmin(ppk,.99999) #Subtract small constant to the largest redefined pp-value, same reason

Z=sum(qnorm(ppk))/sqrt(n-k)

return(pnorm(Z))

} #End function dropk

#7.2 Apply function, in loop with increasing number of exclusions, to full p-curve

#Empty vectors for results

droplow.r=droplow.33=drophigh.r=drophigh.33=c()

#Loop over full p-curves

for (i in 0:(round(ksig/2)-1))

{

#Drop the lowest k studies in terms of respective overall test

#Right skew

droplow.r= c(droplow.r, dropk(pp=ppr,k=i,droplow=1))

drophigh.r=c(drophigh.r, dropk(pp=ppr,k=i,droplow=0))

#Power of 33%

droplow.33=c(droplow.33, dropk(pp=pp33,k=i,droplow=1))

drophigh.33=c(drophigh.33, dropk(pp=pp33,k=i,droplow=0))

}

#Half p-curves

if (khalf>0)

{

droplow.halfr=drophigh.halfr=c()

for (i in 0:(round(khalf/2)-1))

{

#Drop the lowest k studies in terms of respective overall test

droplow.halfr= c(droplow.halfr, dropk(pp=ppr.half,k=i,droplow=1))

drophigh.halfr=c(drophigh.halfr, dropk(pp=ppr.half,k=i,droplow=0))

} #End loop

}#End if that runs calculations only if khalf>0

#7.3 FUNCTION THAT DOES THE PLOT OF RESULTS

plotdrop=function(var,col)

{

k=length(var)

#Plot the dots

plot(0:(k-1),var,xlab="",ylab="",type="b",yaxt="n",xaxt="n",main="",

cex.main=1.15,ylim=c(0,1),col=col)

#Add marker in results with 0 drops

points(0,var[1],pch=19,cex=1.6)

#Red line at p=.05

abline(h=.05,col="red")

#Y-axis value labels

axis(2,c(.05,2:9/10),labels=c('.05','.2','.3','.4','.5','6','7','.8','.9'),las=1,cex.axis=1.5)

axis(1,c(0:(k-1)),las=1,cex.axis=1.4)

}

######################################################################################

# 9. Effect Estimation ###############################################################

######################################################################################

if (effect.estimation == TRUE){

# Define ci.to.t function

ci.to.t = function(TE, lower, upper, n){

z.to.d = function(z, n){

d = (2*z)/sqrt(n)

return(abs(d))

}

ci.to.p = function(est, lower, upper){

SE = (upper-lower)/(2*1.96)

z = abs(est/SE)

p = exp(-0.717*z - 0.416*z^2)

return(p)

}

d.to.t = function(d, n){

df = n-2

t = (d*sqrt(df))/2

return(t)

}

p = ci.to.p(TE, lower, upper)

z = abs(qnorm(p/2))

d = z.to.d(z, n)

t = d.to.t(d, n)

return(t)

}

#Function 13 - loss function

loss=function(t_obs,df_obs,d_est) {

#1.Convert all ts to the same sign (for justification see Supplement 5)

t_obs=abs(t_obs)

#2 Compute p-values

p_obs=2*(1-pt(t_obs,df=df_obs))

#3 Keep significant t-values and corresponding df.

t.sig=subset(t_obs,p_obs<.05)

df.sig=subset(df_obs,p_obs<.05)

#4.Compute non-centrality parameter implied by d_est and df_obs

#df+2 is total N.

#Becuase the noncentrality parameter for the student distribution is ncp=sqrt(n/2)*d,

#we add 2 to d.f. to get N, divide by 2 to get n, and by 2 again for ncp, so -->df+2/4

ncp_est=sqrt((df.sig+2)/4)*d_est

#5.Find critical t-value for p=.05 (two-sided)

#this is used below to compute power, it is a vector as different tests have different dfs

#and hence different critical values

tc=qt(.975,df.sig)

#4.Find power for ncp given tc, again, this is a vector of implied power, for ncp_est, for each test

power_est=1-pt(tc,df.sig,ncp_est)

#5.Compute pp-values

#5.1 First get the overall probability of a t>tobs, given ncp

p_larger=pt(t.sig,df=df.sig,ncp=ncp_est)

#5.2 Now, condition on p<.05

ppr=(p_larger-(1-power_est))/power_est #this is the pp-value for right-skew

#6. Compute the gap between the distribution of observed pp-values and a uniform distribution 0,1

KSD=ks.test(ppr,punif)$statistic #this is the D statistic outputted by the KS test against uniform

return(KSD)

}

if(missing(N)){

stop("If 'effect.estimation=TRUE', argument 'N' must be provided.")

}

if (length(N) != length(metaobject$TE)){

stop("N must be of same length as the number of studies contained in x.")

}

lower = metaobject$TE - (metaobject$seTE*1.96)

upper = metaobject$TE + (metaobject$seTE*1.96)

t_obs = ci.to.t(metaobject$TE, lower, upper, N)

df_obs = N-2

#Results will be stored in these vectors, create them first

loss.all=c()

di=c()

#Compute loss for effect sizes between d=c(dmin,dmax) in steps of .01

for (i in 0:((dmax-dmin)*100))

{

d=dmin+i/100 #effect size being considered

di=c(di,d) #add it to the vector (kind of silly, but kept for symmetry)

options(warn=-1) #turn off warning becuase R does not like its own pt() function!

loss.all=c(loss.all,loss(df_obs=df_obs,t_obs=t_obs,d_est=d))

#apply loss function so that effect size, store result

options(warn=0) #turn warnings back on

}

#find the effect leading to smallest loss in that set, that becomes the starting point in the optimize command

imin=match(min(loss.all),loss.all) #which i tested effect size lead to the overall minimum?

dstart=dmin+imin/100 #convert that i into a d.

#optimize around the global minimum

dhat=optimize(loss,c(dstart-.1,dstart+.1), df_obs=df_obs,t_obs=t_obs)

options(warn=-0)

#Plot results

plot(di,loss.all,xlab="Effect size\nCohen-d", ylab="Loss (D stat in KS test)",ylim=c(0,1), main="How well does each effect size fit? (lower is better)")

points(dhat$minimum,dhat$objective,pch=19,col="red",cex=2)

text(dhat$minimum,dhat$objective-.08,paste0("p-curve's estimate of effect size:\nd=",round(dhat$minimum,3)),col="red")

}

######################################################################################

# 10. Prepare Results for Return #####################################################

######################################################################################

# Get results

main.results = round(main.results, 3)

ktotal = round(main.results[1]) # Get the total number of inserted TEs

k.sign = round(main.results[2]) # Get the total number of significant TEs

k.025 = round(main.results[3]) # Get the number of p<0.25 TEs

skew.full.z = main.results[4] # Get the Z-score for the full curve skewness test

skew.full.p = main.results[5] # Get the p-value for the full curve skewness test

flat.full.z = main.results[6] # Get the Z-score for the full curve flatness test

flat.full.p = main.results[7] # Get the p-value for the full curve flatness test

skew.half.z = main.results[8] # Get the Z-score for the half curve skewness test

skew.half.p = main.results[9] # Get the p-value for the half curve skewness test

flat.half.z = main.results[10] # Get the Z-score for the half curve flatness test

flat.half.p = main.results[11] # Get the p-value for the half curve flatness test

skew.binomial.p = round(binomial[3], 3) # Get the skewness binomial p-value

flat.binomial.p = round(binomial[4], 3) # Get the flatness binomial p-value

# Make data.frame

skewness = c(skew.binomial.p, skew.full.z, skew.full.p, skew.half.z, skew.half.p)

flatness = c(flat.binomial.p, flat.full.z, flat.full.p, flat.half.z, flat.half.p)

colnames.df = c("pBinomial", "zFull", "pFull", "zHalf", "pHalf")

rownames.df = c("Right-skewness test", "Flatness test")

pcurveResults = rbind(skewness, flatness)

colnames(pcurveResults) = colnames.df

rownames(pcurveResults) = rownames.df

# Power results

power_results = round(power_results, 3)

powerEstimate = power_results[2]

powerLower = power_results[1]

powerUpper = power_results[3]

Power = as.data.frame(cbind(powerEstimate, powerLower, powerUpper))

rownames(Power) = ""

# Presence and absence of evidential value

# - If the half p-curve test is right-skewed with p<.05 or both the half and full test

# are right-skewed with p<.1, then p-curve analysis indicates the presence of evidential value

# - Evidential value is inadequate or absent if the 33% power test is p<.05 for the full p-curve

# or both the half p-curve and binomial 33% power test are p<.1

if (skew.half.p < 0.05 | (skew.half.p < 0.1 & skew.full.p < 0.1)){

presence.ev = "yes"

} else {

presence.ev = "no"

}

if (flat.full.p < 0.05 | (flat.half.p < 0.1 & flat.binomial.p < 0.1)){

absence.ev = "yes"

} else {

absence.ev = "no"

}

# Plot Data

PlotData = round(table_figure, 3)

# Input Data

table_calc[,1] = NULL

colnames(table_calc) = c("p", "ppSkewFull", "ppSkewHalf", "ppFlatFull", "ppFlatHalf", "zSkewFull", "zSkewHalf",

"zFlatFull", "zFlatHalf")

Input = cbind(metaobject$TE, round(table_calc,3))

rownames(Input) = paste(1:length(metaobject$TE), metaobject$studlab)

colnames(Input)[1] = "TE"

if (effect.estimation==TRUE){

dEstimate = round(dhat$minimum, 3)

return.list = list("pcurveResults" = pcurveResults,

"Power" = Power,

"PlotData" = PlotData,

"Input" = Input,

"EvidencePresent" = presence.ev,

"EvidenceAbsent" = absence.ev,

"kInput" = ktot,

"kAnalyzed" = k.sign,

"kp0.25" = k.025,

"dEstimate" = dEstimate,

"I2" = metaobject$I2,

"class.meta.object" = class(metaobject)[1])

class(return.list) = c("pcurve", "effect.estimation")

} else {

return.list = list("pcurveResults" = pcurveResults,

"Power" = Power,

"PlotData" = PlotData,

"Input" = Input,

"EvidencePresent" = presence.ev,

"EvidenceAbsent" = absence.ev,

"kInput" = ktot,

"kAnalyzed" = k.sign,

"kp0.25" = k.025,

"I2" = metaobject$I2,

"class.meta.object" = class(metaobject)[1])

class(return.list) = c("pcurve", "no.effect.estimation")

}

cat(" ", "\n")

invisible(return.list)

return.list

}
