# Supplementary material for: Efficacy of dihydroartemisinin-piperaquine versus artemether-lumefantrine for the treatment of uncomplicated Plasmodium falciparum malaria among children in Africa: a systematic review and meta-analysis of randomized control trials
Source: Malar J. 2021 Aug 12;20:340. doi: 10.1186/s12936-021-03873-1 (PMC8359548; doi:10.1186/s12936-021-03873-1)
Supplement: Supplementary file 4 — Additional file 4. Characteristics of included studies. [file 12936_2021_3873_MOESM4_ESM.docx]

Additional file S 3: Characteristics of excluded studies

| **No** | **Studies** | **Reason for exclusion** |
| --- | --- | --- |
|  | Adam-2010-SUD [115] | Both children and adults were enrolled in this trial. Children’s outcome didn’t report. |
|  | Conrad-2014-UGA [144] | The outcomes were not relevant for this study. |
|  | Creek-2010-UGA[137] | The outcomes were reported in another study. |
|  | Dama-2018-MAL [38] | Both children and adults were enrolled in this trial. Children’s outcome didn’t report. |
|  | Davlantes-2018-ANG [126] | Both children and adults were enrolled in this trial. Children’s outcome didn’t report. |
|  | Diallo-2020-SEN [122] | Both children and adults were enrolled in this trial. Children’s outcome didn’t report. |
|  | Funck -2019 [39] | The outcomes were not relevant for this study. |
|  | Green-2016 [145] | The outcomes were not relevant for this study. |
|  | Ishengoma-2019-TAN [146] | The outcomes were not relevant for this study. |
|  | Kakolwa-2018-TAN [147] | The outcomes were not relevant for this study. |
|  | Kakuru-2013-UGA [148] | The outcomes were reported in another study. |
|  | Katrak-2009-UGA [149] | The outcomes were reported in another study. |
|  | Menan-2011- AFR [144] | Both children and adults were enrolled in this trial. Children’s outcome didn’t report. |
|  | Omondi-2019-KEN [150] | The outcomes were not relevant for this study. |
|  | Plucinski-2015-ANG [114] | Non-randomized trial. |
|  | Plucinski-2017-ANG [113] | Non-randomized trial. |
|  | Sylla-2013-SEN [61] | The outcomes were not relevant for this study. |
|  | Sow-2016-SEN [60] | Both children and adults were enrolled in this trial. Children’s outcome didn’t report. |
|  | Tylor-2017-UGA [151] | The outcomes were not relevant for this study. |
|  | Van-2020-MCT [152] | The outcomes were not relevant for this study. |
|  | Verret-2009-UGA [153] | The outcomes were reported in another study. |
|  | Warsame-2019-SOM [116] | Both children and adults were enrolled in this trial. Children’s outcome didn’t report. |
|  | Yavo-2011-SSA [43] | Both children and adults were enrolled in this trial. Children’s outcome didn’t report. |
|  | Yeka-2013-UGA [154] | The outcomes were reported in another study. |
