# Supplementary material for: Efficacy of dihydroartemisinin-piperaquine versus artemether-lumefantrine for the treatment of uncomplicated Plasmodium falciparum malaria among children in Africa: a systematic review and meta-analysis of randomized control trials
Source: Malar J. 2021 Aug 12;20:340. doi: 10.1186/s12936-021-03873-1 (PMC8359548; doi:10.1186/s12936-021-03873-1)
Supplement: Supplementary file 5 — Additional file 5. Meta-regression of PCR-unadjusted treatment failure at day 28, association between age of the children and treatment failure. [file 12936_2021_3873_MOESM5_ESM.docx]

Additional file S4: Characteristics of included studies

| S. No | | Study ID | | | Study design | | | Study setting and period | | | | | | Transmission | | Follow up | | | Subjects | | | | | | | | Patient important outcome | DHA-PQ | AL |
| --- | --- | --- | --- | --- | --- | --- | --- | --- | --- | --- | --- | --- | --- | --- | --- | --- | --- | --- | --- | --- | --- | --- | --- | --- | --- | --- | --- | --- | --- |
|  |  |  |  |  |  |  |  |  |  |  |  |  |  |  |  |  |  |  | **Number of participants** | | | | | | **Inclusion age** | |  |  |  |
|  |  |  |  |  |  |  |  |  |  |  |  |  |  |  |  |  |  |  | **DHA-PQ** | | | **AL** | | |  | |  |  |  |
| 1 | | **Kamya-2007-UGA [97]** | | | Single-blind, RCT | | | Rural health center, March,2006-July, 2006 | | | | | | High transmission | | 42 days | | | 253 | | | 256 | | | 6 months-10 years | | Loss to follow up | 0 | 0 |
|  | | | | | | | | | | | | | | | | | | | | | | | | | | | ETF | 0 | 0 |
|  |  |  |  |  |  |  |  |  |  |  |  |  |  |  |  |  |  |  |  |  |  |  |  |  |  |  | LCF | 19 | 30 |
|  |  |  |  |  |  |  |  |  |  |  |  |  |  |  |  |  |  |  |  |  |  |  |  |  |  |  | LPF | 73 | 89 |
|  |  |  |  |  |  |  |  |  |  |  |  |  |  |  |  |  |  |  |  |  |  |  |  |  |  |  | ACPR | 117 | 89 |
|  |  |  |  |  |  |  |  |  |  |  |  |  |  |  |  |  |  |  |  |  |  |  |  |  |  |  | Fever clearance at day 1 ^b^ | 137 | 137 |
|  |  |  |  |  |  |  |  |  |  |  |  |  |  |  |  |  |  |  |  |  |  |  |  |  |  |  | Fever clearance at day 2 | 66 | 72 |
|  |  |  |  |  |  |  |  |  |  |  |  |  |  |  |  |  |  |  |  |  |  |  |  |  |  |  | Fever clearance at day 3 | 52 | 57 |
|  |  |  |  |  |  |  |  |  |  |  |  |  |  |  |  |  |  |  |  |  |  |  |  |  |  |  | Parasite clearance at day 2 | 1 | 2 |
|  |  |  |  |  |  |  |  |  |  |  |  |  |  |  |  |  |  |  |  |  |  |  |  |  |  |  | Parasite clearance at day 3 | 0 | 0 |
| 2 | | **Zongo-2007-BNF** [98] | | | Single blind RCT | | | Government health dispensaries,  August 2006- January 2007 | | | | | High transmission | | | 42 days | | | 196 | | | 197 | | | 6 months-10 years | | Withdrawn | 24 | 21 |
|  |  |  |  |  |  |  |  |  |  |  |  |  |  |  |  |  |  |  |  |  |  |  |  |  |  |  | ETF | 2 | 2 |
|  |  |  |  |  |  |  |  |  |  |  |  |  |  |  |  |  |  |  |  |  |  |  |  |  |  |  | LCF | 8 | 34 |
|  |  |  |  |  |  |  |  |  |  |  |  |  |  |  |  |  |  |  |  |  |  |  |  |  |  |  | LPF | 3 | 19 |
|  |  |  |  |  |  |  |  |  |  |  |  |  |  |  |  |  |  |  |  |  |  |  |  |  |  |  | ACPR | 159 | 121 |
|  | | | | | | | | | | | | | | | | | | | | | | | | | | |  |  |  |
|  |  |  |  |  |  |  |  |  |  |  |  |  |  |  |  |  |  |  |  |  |  |  |  |  |  |  |  |  |  |
|  |  |  |  |  |  |  |  |  |  |  |  |  |  |  |  |  |  |  |  |  |  |  |  |  |  |  | Fever on day 1 | 70 | 91 |
|  |  |  |  |  |  |  |  |  |  |  |  |  |  |  |  |  |  |  |  |  |  |  |  |  |  |  | Fever on day 2 | 23 | 26 |
|  |  |  |  |  |  |  |  |  |  |  |  |  |  |  |  |  |  |  |  |  |  |  |  |  |  |  | Fever on day 3 | 17 | 15 |
|  |  |  |  |  |  |  |  |  |  |  |  |  |  |  |  |  |  |  |  |  |  |  |  |  |  |  | Parasite clearance at day 2 | 6 | 5 |
|  |  |  |  |  |  |  |  |  |  |  |  |  |  |  |  |  |  |  |  |  |  |  |  |  |  |  | Parasite clearance at day 3 | 0 | 0 |
| 3 | | **Mens-2008-KEN** [111] | | | Open label RCT | | | Health center,  Apr 2007 to Jul 2007 | | | | | High transmission | | | 28 days | | | 73 | | | 73 | | | 6 months-12 years | | Withdrawn | 6 | 6 |
|  |  |  |  |  |  |  |  |  |  |  |  |  |  |  |  |  |  |  |  |  |  |  |  |  |  |  | Re-infection at day 28 | 0 | 1 |
|  |  |  |  |  |  |  |  |  |  |  |  |  |  |  |  |  |  |  |  |  |  |  |  |  |  |  | Recrudescence at day 28 | 0 | 0 |
|  | | | | | | | | | | | | | | | | | | | | | | | | | | |  |  |  |
|  |  |  |  |  |  |  |  |  |  |  |  |  |  |  |  |  |  |  |  |  |  |  |  |  |  |  | Fever clearance at day 1 ^b^ | 10 | 6 |
|  |  |  |  |  |  |  |  |  |  |  |  |  |  |  |  |  |  |  |  |  |  |  |  |  |  |  | Fever clearance at day 2 | 4 | 12 |
|  |  |  |  |  |  |  |  |  |  |  |  |  |  |  |  |  |  |  |  |  |  |  |  |  |  |  | Fever clearance at day 3 | 3 | 2 |
|  |  |  |  |  |  |  |  |  |  |  |  |  |  |  |  |  |  |  |  |  |  |  |  |  |  |  | Parasite clearance at day 2 | 1 | 0 |
| 4 | | **Yeka-2008-UGA [99]** | | | Single-blind, RCT | | | Health center, August 2006- April 2007 | | | | | N/A | | | 42 days | | | 234 | | | 227 | | | 6 months-10 years | | Los to follow up | 3 | 3 |
|  |  |  |  |  |  |  |  |  |  |  |  |  |  |  |  |  |  |  |  |  |  |  |  |  |  |  | ETF | 0 | 1 |
|  |  |  |  |  |  |  |  |  |  |  |  |  |  |  |  |  |  |  |  |  |  |  |  |  |  |  | LCF | 9 | 23 |
|  |  |  |  |  |  |  |  |  |  |  |  |  |  |  |  |  |  |  |  |  |  |  |  |  |  |  | LPF | 17 | 41 |
|  | | | | | | | | | | | | | | | | | | | | | | | | | | | ACPR | 186 | 131 |
|  |  |  |  |  |  |  |  |  |  |  |  |  |  |  |  |  |  |  |  |  |  |  |  |  |  |  | Fever clearance at day 1 ^b^ | 117 | 133 |
|  |  |  |  |  |  |  |  |  |  |  |  |  |  |  |  |  |  |  |  |  |  |  |  |  |  |  | Fever clearance at day 2 | 44 | 37 |
|  |  |  |  |  |  |  |  |  |  |  |  |  |  |  |  |  |  |  |  |  |  |  |  |  |  |  | Fever clearance at day 3 | 22 | 22 |
|  |  |  |  |  |  |  |  |  |  |  |  |  |  |  |  |  |  |  |  |  |  |  |  |  |  |  | Parasite clearance at day 2 | 7 | 5 |
|  |  |  |  |  |  |  |  |  |  |  |  |  |  |  |  |  |  |  |  |  |  |  |  |  |  |  | Parasite clearance at day 3 | 0 | 0 |
| 5 | | **Bassat-2009-AFR** [101] | | | Open-label, RCT | | | Four rural sites and one peri-urban site,  August 2005 and July 2006. | | | | | | | | Mesoendemic | | | 1038 | | | 510 | | | 6–59 months | | Withdrawn | 61 | 36 |
|  |  |  |  |  |  |  |  |  |  |  |  |  |  |  |  |  |  |  |  |  |  |  |  |  |  |  | Treatment failure at day 28 | 77 | 88 |
|  | | | | | | | | | | | | | | | | | | | | | | | | | | | Recrudescence at day 28 | 14 | 11 |
|  |  |  |  |  |  |  |  |  |  |  |  |  |  |  |  |  |  |  |  |  |  |  |  |  |  |  | Treatment failure at day 42 | 200 | 147 |
|  |  |  |  |  |  |  |  |  |  |  |  |  |  |  |  |  |  |  |  |  |  |  |  |  |  |  | Recrudescence at day 42 | 41 | 16 |
| 6 | | **Arinaitwe-2009-UGA** [100] | | | Open-label RCT | | | Local antenatal clinics in Tororo, August 2007-July 2008 | | | | | High transmission | | | 63 days | | | 119 | | | 111 | | | 6 weeks- 12 months | | No treatment outcome | 6 | 5 |
|  | | | | | | | | | | | | | | | | | | | | | | | | | | | ETF | 0 | 0 |
|  |  |  |  |  |  |  |  |  |  |  |  |  |  |  |  |  |  |  |  |  |  |  |  |  |  |  | LCF at day 28 | 13 | 45 |
|  |  |  |  |  |  |  |  |  |  |  |  |  |  |  |  |  |  |  |  |  |  |  |  |  |  |  | LPF at day 28 | 26 | 64 |
|  |  |  |  |  |  |  |  |  |  |  |  |  |  |  |  |  |  |  |  |  |  |  |  |  |  |  | ACPR at day 28 | 306 | 205 |
|  |  |  |  |  |  |  |  |  |  |  |  |  |  |  |  |  |  |  |  |  |  |  |  |  |  |  | PCR unadjusted Rx failure at day 42 | 16 | 33 |
|  |  |  |  |  |  |  |  |  |  |  |  |  |  |  |  |  |  |  |  |  |  |  |  |  |  |  | PCR adjusted Rx failure at day 42 | 0 | 0 |
|  |  |  |  |  |  |  |  |  |  |  |  |  |  |  |  |  |  |  |  |  |  |  |  |  |  |  | PCR unadjusted Rx failure at day 63 | 46 | 110 |
|  |  |  |  |  |  |  |  |  |  |  |  |  |  |  |  |  |  |  |  |  |  |  |  |  |  |  | PCR adjusted Rx failure at day 63 | 8 | 4 |
|  |  |  |  |  |  |  |  |  |  |  |  |  |  |  |  |  |  |  |  |  |  |  |  |  |  |  | Fever clearance at day 1 ^b^ | 138 | 163 |
|  |  |  |  |  |  |  |  |  |  |  |  |  |  |  |  |  |  |  |  |  |  |  |  |  |  |  | Fever clearance at day 2 | 13 | 17 |
|  |  |  |  |  |  |  |  |  |  |  |  |  |  |  |  |  |  |  |  |  |  |  |  |  |  |  | Fever clearance at day 3 | 9 | 12 |
|  |  |  |  |  |  |  |  |  |  |  |  |  |  |  |  |  |  |  |  |  |  |  |  |  |  |  | Parasite clearance at day 2 | 12 | 22 |
|  |  |  |  |  |  |  |  |  |  |  |  |  |  |  |  |  |  |  |  |  |  |  |  |  |  |  | Parasite clearance at day 3 | 1 | 0 |
| 7 | | **Borrmann-2011 –KEN [109]** | | | Not described, RCT | | | Pingilikani study site,  September 2005 to April 2008 | | | | | Perennial transmission | | | 84 days | | | 233 | | | 241 | | | 6–59 months | | withdrawn | 40 | 25 |
|  |  |  |  |  |  |  |  |  |  |  |  |  |  |  |  |  |  |  |  |  |  |  |  |  |  |  | ETF | 2 | 2 |
|  | | | | | | | | | | | | | | | | | | | | | | | | | | | Parasite clearance at day 1 | 151 | 214 |
|  |  |  |  |  |  |  |  |  |  |  |  |  |  |  |  |  |  |  |  |  |  |  |  |  |  |  | Parasite clearance at day 2 | 10 | 26 |
|  |  |  |  |  |  |  |  |  |  |  |  |  |  |  |  |  |  |  |  |  |  |  |  |  |  |  | Parasite clearance at day 3 | 0 | 0 |
| 8 | | **Nambozi-2011- ZAM** [37] | | | Open-label, RCT | | | Peri-urban health centers,  September 2005 and May 2006 | | | | | Mesoendemic | | | 42 days | | | 203 | | | 101 | | | 6-59 months | | Withdrawn | 11 | 11 |
|  | | | | | | | | | | | | | | | | | | | | | | | | | | | LCF PCR un adjusted at day 28 | 6 | 10 |
|  |  |  |  |  |  |  |  |  |  |  |  |  |  |  |  |  |  |  |  |  |  |  |  |  |  |  | LPF PCR un adjusted at day 28 | 9 | 13 |
|  |  |  |  |  |  |  |  |  |  |  |  |  |  |  |  |  |  |  |  |  |  |  |  |  |  |  | ACPR PCR un adjusted at day 28 | 177 | 67 |
|  |  |  |  |  |  |  |  |  |  |  |  |  |  |  |  |  |  |  |  |  |  |  |  |  |  |  | LCF PCR-adjusted at day 28 | 0 | 3 |
|  |  |  |  |  |  |  |  |  |  |  |  |  |  |  |  |  |  |  |  |  |  |  |  |  |  |  | LPF PCR-adjusted at day 28 | 9 | 3 |
|  |  |  |  |  |  |  |  |  |  |  |  |  |  |  |  |  |  |  |  |  |  |  |  |  |  |  | ACPR PCR-adjusted at day 28 | 183 | 84 |
|  |  |  |  |  |  |  |  |  |  |  |  |  |  |  |  |  |  |  |  |  |  |  |  |  |  |  | LCF PCR un adjusted at day 42 | 11 | 13 |
|  |  |  |  |  |  |  |  |  |  |  |  |  |  |  |  |  |  |  |  |  |  |  |  |  |  |  | LPF PCR un adjusted at day 42 | 31 | 20 |
|  |  |  |  |  |  |  |  |  |  |  |  |  |  |  |  |  |  |  |  |  |  |  |  |  |  |  | ACPR PCR un adjusted at day 42 | 150 | 57 |
|  |  |  |  |  |  |  |  |  |  |  |  |  |  |  |  |  |  |  |  |  |  |  |  |  |  |  | LCF PCR-adjusted at day 42 | 4 | 3 |
|  |  |  |  |  |  |  |  |  |  |  |  |  |  |  |  |  |  |  |  |  |  |  |  |  |  |  | LPF PCR adjusted at day 42 | 9 | 3 |
|  |  |  |  |  |  |  |  |  |  |  |  |  |  |  |  |  |  |  |  |  |  |  |  |  |  |  | ACPR PCR adjusted at day 42 | 179 | 84 |
| 9 | | **4ABC-2011-AFR** [80] | | | Open label, RCT | | | Rural, urban or health facilities, 9 July 2007 and 19 June 2009 | | | | | Mesoendemic, perennial and high transmission | | | 63 days | | | 1475 | | | 1226 | | | 6 to 59 months | | Withdrawn | 103 | 66 |
|  |  |  |  |  |  |  |  |  |  |  |  |  |  |  |  |  |  |  |  |  |  |  |  |  |  |  | ETF | 2 | 0 |
|  |  |  |  |  |  |  |  |  |  |  |  |  |  |  |  |  |  |  |  |  |  |  |  |  |  |  | New infections at day 28 | 98 | 268 |
|  | | | | | | | | | | | | | | | | | | | | | | | | | | | Recrudescence at day 28 | 22 | 41 |
|  |  |  |  |  |  |  |  |  |  |  |  |  |  |  |  |  |  |  |  |  |  |  |  |  |  |  | Death up to day 63 | 1 | 3 |
|  |  |  |  |  |  |  |  |  |  |  |  |  |  |  |  |  |  |  |  |  |  |  |  |  |  |  | New infections at day 63 | 277 | 166 |
|  |  |  |  |  |  |  |  |  |  |  |  |  |  |  |  |  |  |  |  |  |  |  |  |  |  |  | Recrudescence at day 63 | 23 | 8 |
|  |  |  |  |  |  |  |  |  |  |  |  |  |  |  |  |  |  |  |  |  |  |  |  |  |  |  | Day 28: no PCR results | 12 | 25 |
|  |  |  |  |  |  |  |  |  |  |  |  |  |  |  |  |  |  |  |  |  |  |  |  |  |  |  | Day 63: no PCR result | 83 | 67 |
| 10 | | **Agarwal -2013-KEN [35]** | | | An open label RCT | | | District hospital, October 2010 to August 2011 | | | | | High transmission | | | 42 days | | | 137 | | | 137 | | | 6 to 59 months | | Withdrawn | 24 | 26 |
|  |  |  |  |  |  |  |  |  |  |  |  |  |  |  |  |  |  |  |  |  |  |  |  |  |  |  | LCF at day 28 | 4 | 13 |
|  |  |  |  |  |  |  |  |  |  |  |  |  |  |  |  |  |  |  |  |  |  |  |  |  |  |  | LPF at day 28 | 16 | 32 |
|  | | | | | | | | | | | | | | | | | | | | | | | | | | | LCF at day 42 | 11 | 21 |
|  |  |  |  |  |  |  |  |  |  |  |  |  |  |  |  |  |  |  |  |  |  |  |  |  |  |  | LPF at day 42 | 41 | 41 |
|  |  |  |  |  |  |  |  |  |  |  |  |  |  |  |  |  |  |  |  |  |  |  |  |  |  |  | Recrudescence at day 28 | 1 | 3 |
|  |  |  |  |  |  |  |  |  |  |  |  |  |  |  |  |  |  |  |  |  |  |  |  |  |  |  | Recrudescence at day 42 | 4 | 4 |
|  |  |  |  |  |  |  |  |  |  |  |  |  |  |  |  |  |  |  |  |  |  |  |  |  |  |  | Fever clearance at day 2 ^b^ | 5 | 4 |
|  |  |  |  |  |  |  |  |  |  |  |  |  |  |  |  |  |  |  |  |  |  |  |  |  |  |  | Fever clearance at day 3 | 0 | 1 |
| 11 | **Meremikwu-2013-NIG** [108] | | | Open label, RCT | | | | N/A | | | | N/A | | | | | 63 days | | 77 | | | | 92 | | 6 to 59months | | Withdrawn | 5 | 5 |
|  |  |  |  |  |  |  |  |  |  |  |  |  |  |  |  |  |  |  |  |  |  |  |  |  |  |  | PCR unadjusted treatment failure at day 28 | 5 | 3 |
|  |  |  |  |  |  |  |  |  |  |  |  |  |  |  |  |  |  |  |  |  |  |  |  |  |  |  | PCR adjusted treatment failure at day 28 | 0 | 2 |
|  | | | | | | | | | | | | | | | | | | | | | | | | | | | PCR unadjusted treatment failure at day 63 | 5 | 6 |
|  |  |  |  |  |  |  |  |  |  |  |  |  |  |  |  |  |  |  |  |  |  |  |  |  |  |  | PCR adjusted treatment failure at day 63 | 0 | 3 |
| 12 | | **SAWA-2013 –KEN [102]** | | | Single-blind, RCT | | | Community setting,  April to June 2009 | | | | | Moderate transmission | | | 42 days | | | 145 | | | 153 | | | 6 months to 10 years | | Withdrawn | 11 | 8 |
|  |  |  |  |  |  |  |  |  |  |  |  |  |  |  |  |  |  |  |  |  |  |  |  |  |  |  | Recrudescence at day 28 | 0 | 2 |
|  | | | | | | | | | | | | | | | | | | | | | | | | | | | New infection at day 28 | 0 | 8 |
|  |  |  |  |  |  |  |  |  |  |  |  |  |  |  |  |  |  |  |  |  |  |  |  |  |  |  | Undetermined PCR at day 28 | 0 | 0 |
|  |  |  |  |  |  |  |  |  |  |  |  |  |  |  |  |  |  |  |  |  |  |  |  |  |  |  | Recrudescence at day42 | 0 | 4 |
|  |  |  |  |  |  |  |  |  |  |  |  |  |  |  |  |  |  |  |  |  |  |  |  |  |  |  | New infection at day 42 | 3 | 23 |
|  |  |  |  |  |  |  |  |  |  |  |  |  |  |  |  |  |  |  |  |  |  |  |  |  |  |  | Undetermined PCR at day 42 | 2 | 3 |
|  |  |  |  |  |  |  |  |  |  |  |  |  |  |  |  |  |  |  |  |  |  |  |  |  |  |  | Parasite clearance at day 2 | 10 | 5 |
|  |  |  |  |  |  |  |  |  |  |  |  |  |  |  |  |  |  |  |  |  |  |  |  |  |  |  | Parasite clearance at day 3 | 1 | 0 |
| 13 | | **Muhindo-2014-UGA [92]** | | | Open-label, RCT | | | Post-natal clinic at Tororo District Hospital, October 2011 to December 2012 | | | | | High-transmission | | | 28 days | | | 106 | | | 96 | | | 6 weeks to 12 months | | Withdrawn | 13 | 16 |
|  |  |  |  |  |  |  |  |  |  |  |  |  |  |  |  |  |  |  |  |  |  |  |  |  |  |  | ETF | 1 | 0 |
|  |  |  |  |  |  |  |  |  |  |  |  |  |  |  |  |  |  |  |  |  |  |  |  |  |  |  | LCF | 7 | 74 |
|  |  |  |  |  |  |  |  |  |  |  |  |  |  |  |  |  |  |  |  |  |  |  |  |  |  |  | LPF | 22 | 137 |
|  |  |  |  |  |  |  |  |  |  |  |  |  |  |  |  |  |  |  |  |  |  |  |  |  |  |  | ACPR | 311 | 189 |
|  |  |  |  |  |  |  |  |  |  |  |  |  |  |  |  |  |  |  |  |  |  |  |  |  |  |  | Fever clearance at day 1 | 65 | 124 |
|  | | | | | | | | | | | | | | | | | | | | | | | | | | | Fever clearance at day 2 | 11 | 8 |
|  |  |  |  |  |  |  |  |  |  |  |  |  |  |  |  |  |  |  |  |  |  |  |  |  |  |  | Fever clearance at day 3 | 7 | 7 |
|  |  |  |  |  |  |  |  |  |  |  |  |  |  |  |  |  |  |  |  |  |  |  |  |  |  |  | Parasite clearance at day 1 | 181 | 269 |
|  |  |  |  |  |  |  |  |  |  |  |  |  |  |  |  |  |  |  |  |  |  |  |  |  |  |  | Parasite clearance at day 2 | 20 | 23 |
|  |  |  |  |  |  |  |  |  |  |  |  |  |  |  |  |  |  |  |  |  |  |  |  |  |  |  | Parasite clearance at day 3 | 1 | 0 |
| 14 | | **Ogutu-2014-KEN** [42] | | | Open-label, RCT | | | Nyando District hospital, March, 2010- 30 November, 2011 | | | | | Not described | | | 42 days | | | 227 | | | 227 | | | 6 to 59 months | | Withdrawn | 2 | 4 |
|  |  |  |  |  |  |  |  |  |  |  |  |  |  |  |  |  |  |  |  |  |  |  |  |  |  |  | PCR uncorrected treatment failure at day 28 | 28 | 43 |
|  | | | | | | | | | | | | | | | | | | | | | | | | | | | PCR corrected treatment failure at day 28 | 2 | 5 |
|  |  |  |  |  |  |  |  |  |  |  |  |  |  |  |  |  |  |  |  |  |  |  |  |  |  |  | PCR uncorrected treatment failure at day 42 | 67 | 73 |
|  |  |  |  |  |  |  |  |  |  |  |  |  |  |  |  |  |  |  |  |  |  |  |  |  |  |  | PCR corrected treatment failure at day 42 | 3 | 7 |
| 15 | | **Onyamboko-2014-DRC** [103] | | | Open label, RCT | | | Urban district of Kinshasa (DRC) (Hospitals), September 2011 and November 2012 | | | | | Intense and perennial | | | 42 days | | | 228 | | | 228 | | | 3 to 59 months | | Withdrawn | 16 | 10 |
|  |  |  |  |  |  |  |  |  |  |  |  |  |  |  |  |  |  |  |  |  |  |  |  |  |  |  | ETF at day 42 | 1 | 1 |
|  |  |  |  |  |  |  |  |  |  |  |  |  |  |  |  |  |  |  |  |  |  |  |  |  |  |  | LCF at day 42 | 10 | 12 |
|  |  |  |  |  |  |  |  |  |  |  |  |  |  |  |  |  |  |  |  |  |  |  |  |  |  |  | LPF at day 42 | 18 | 52 |
|  |  |  |  |  |  |  |  |  |  |  |  |  |  |  |  |  |  |  |  |  |  |  |  |  |  |  | PCR Unadjusted ACPR at day 28 | 206 | 190 |
|  |  |  |  |  |  |  |  |  |  |  |  |  |  |  |  |  |  |  |  |  |  |  |  |  |  |  | PCR adjusted ACPR at day 28 | 208 | 211 |
|  | | | | | | | | | | | | | | | | | | | | | | | | | | | Fever clearance at day 1 ^b^ | 8 | 11 |
|  |  |  |  |  |  |  |  |  |  |  |  |  |  |  |  |  |  |  |  |  |  |  |  |  |  |  | Fever clearance at day 2 | 1 | 9 |
|  |  |  |  |  |  |  |  |  |  |  |  |  |  |  |  |  |  |  |  |  |  |  |  |  |  |  | Fever clearance at day 3 | 5 | 3 |
| 16 | | **Wanzira-2014- UGA** [143] | | | Open-label, RCT | | | Tororo District Hospital,  February 2009- 2012 | | | | | Very high transmission | | | 28 days | | | 154 | | | 158 | | | 6 weeks to 9 months | | Withdrawn | 25 | 26 |
|  |  |  |  |  |  |  |  |  |  |  |  |  |  |  |  |  |  |  |  |  |  |  |  |  |  |  | ETF | 2 | 15 |
|  |  |  |  |  |  |  |  |  |  |  |  |  |  |  |  |  |  |  |  |  |  |  |  |  |  |  | LCF | 48 | 475 |
|  |  |  |  |  |  |  |  |  |  |  |  |  |  |  |  |  |  |  |  |  |  |  |  |  |  |  | LPF | 182 | 894 |
|  | | | | | | | | | | | | | | | | | | | | | | | | | | | ACPR | 2403 | 1494 |
|  |  |  |  |  |  |  |  |  |  |  |  |  |  |  |  |  |  |  |  |  |  |  |  |  |  |  | Recrudescence at day 63 | 24 | 22 |
| 17 | | **Kakuru-2014-UGA** [110] | | | Not described, RCT | | | District Hospital, August 2007 and April 2008 | | | | | High transmission | | | 28 days | | | 21 | | | 22 | | | 6 weeks -12 months | | Fever clearance at day 1 ^b^ | 46 | 106 |
|  | | | | | | | | | | | | | | | | | | | | | | | | | | | Fever clearance at day 2 | 7 | 16 |
|  |  |  |  |  |  |  |  |  |  |  |  |  |  |  |  |  |  |  |  |  |  |  |  |  |  |  | Fever clearance at day 3 | 5 | 3 |
| 18 | | **Nji-2015-CAM** [106] | | | Open-label, RCT | | | Two distinct ecological regions,  2009 to April 2013 | | | | | Low to moderate transmission | | | 42 days | | | 288 | | | 144 | | | 6 months-10 years | | Withdrawn | 43 | 21 |
|  |  |  |  |  |  |  |  |  |  |  |  |  |  |  |  |  |  |  |  |  |  |  |  |  |  |  | PCR adjusted ETF at day 28 | 2 | 0 |
|  | | | | | | | | | | | | | | | | | | | | | | | | | | |  |  |  |
|  |  |  |  |  |  |  |  |  |  |  |  |  |  |  |  |  |  |  |  |  |  |  |  |  |  |  | PCR adjusted LCF at day 28 | 4 | 1 |
|  |  |  |  |  |  |  |  |  |  |  |  |  |  |  |  |  |  |  |  |  |  |  |  |  |  |  | PCR adjusted LPF at day 28 | 3 | 3 |
|  |  |  |  |  |  |  |  |  |  |  |  |  |  |  |  |  |  |  |  |  |  |  |  |  |  |  | ACPR at day 28 | 236 | 119 |
|  |  |  |  |  |  |  |  |  |  |  |  |  |  |  |  |  |  |  |  |  |  |  |  |  |  |  | PCR unadjusted treatment failure at day 42 | 26 | 11 |
|  |  |  |  |  |  |  |  |  |  |  |  |  |  |  |  |  |  |  |  |  |  |  |  |  |  |  | PCR adjusted treatment failure at day 42 | 9 | 4 |
|  |  |  |  |  |  |  |  |  |  |  |  |  |  |  |  |  |  |  |  |  |  |  |  |  |  |  | SAE | 0 | 1 |
| 19 | | **Ursing-2016-GUB** [107] | | | Open-label, RCT | | | Bandimand Belem Health Centers, November 2012 and July 2015 | | | | | Low to high transmission | | | 42 days | | | 157 | | | 155 | | | 6 months-15 years | | Withdrawn | 17 | 18 |
|  |  |  |  |  |  |  |  |  |  |  |  |  |  |  |  |  |  |  |  |  |  |  |  |  |  |  | ETF | 2 | 6 |
|  |  |  |  |  |  |  |  |  |  |  |  |  |  |  |  |  |  |  |  |  |  |  |  |  |  |  | LCF at day 42 | 0 | 3 |
|  |  |  |  |  |  |  |  |  |  |  |  |  |  |  |  |  |  |  |  |  |  |  |  |  |  |  | New infection at day 42 | 2 | 0 |
| 20 | | **Ebenebe-2018-NIG** [24] | | | Open label, RCT | | | Hospitals and clinics, June 2014 and December 2015 | | | | | High transmission | | | 42 days | | | 347 | | | 324 | | | 6 - 59 months | | Withdrawn | 55 | 44 |
|  |  |  |  |  |  |  |  |  |  |  |  |  |  |  |  |  |  |  |  |  |  |  |  |  |  |  | Parasite clearance at day 1 | 173 | 195 |
|  | |  | | | | | | | | | | | | | | | | | | | | | | | | | Parasite clearance at day 2 | 60 | 76 |
|  |  |  |  |  |  |  |  |  |  |  |  |  |  |  |  |  |  |  |  |  |  |  |  |  |  |  | Parasite clearance at day 3 | 5 | 9 |
|  |  |  |  |  |  |  |  |  |  |  |  |  |  |  |  |  |  |  |  |  |  |  |  |  |  |  | Fever clearance at day 1 ^b^ | 189 | 194 |
|  |  |  |  |  |  |  |  |  |  |  |  |  |  |  |  |  |  |  |  |  |  |  |  |  |  |  | ETF | 2 | 1 |
|  |  |  |  |  |  |  |  |  |  |  |  |  |  |  |  |  |  |  |  |  |  |  |  |  |  |  | Reinfection at day 28 | 11 | 26 |
|  |  |  |  |  |  |  |  |  |  |  |  |  |  |  |  |  |  |  |  |  |  |  |  |  |  |  | Recrudescence at day 28 | 0 | 4 |
|  |  |  |  |  |  |  |  |  |  |  |  |  |  |  |  |  |  |  |  |  |  |  |  |  |  |  | Reinfection at day 42 | 18 | 55 |
|  |  |  |  |  |  |  |  |  |  |  |  |  |  |  |  |  |  |  |  |  |  |  |  |  |  |  | Recrudescence at day 42 | 1 | 7 |
| 21 | | **Grandesso-2018-NIG** [104] | | | Open label, RCT | | | Health center,  7 June 2013 and 22 September 2014 | | Not reported | | | | | 42 days | | | | 221 | | | 221 | | | 6–59 months | | Withdrawn | 11 | 15 |
|  |  |  |  |  |  |  |  |  |  |  |  |  |  |  |  |  |  |  |  |  |  |  |  |  |  |  | Early vomiting | 1 | 0 |
|  |  |  |  |  |  |  |  |  |  |  |  |  |  |  |  |  |  |  |  |  |  |  |  |  |  |  | Reinfection at day 42 | 51 | 64 |
|  |  |  |  |  |  |  |  |  |  |  |  |  |  |  |  |  |  |  |  |  |  |  |  |  |  |  | Undetermined PCR at day 42 | 4 | 10 |
|  | | | | | | | | | | | | | | | | | | | | | | | | | | | PCR adjusted LCF | 1 | 1 |
|  |  |  |  |  |  |  |  |  |  |  |  |  |  |  |  |  |  |  |  |  |  |  |  |  |  |  | PCR adjusted LPF | 2 | 1 |
| 22 | | **Mandara-2018-TAN** [79] | | | Open label, RCT | | | District Hospital and Health Centre, May 2014 and January 2015 | | Low to moderate transmission | | | | | 63 days | | | | 255 | | | 257 | | | 6 months- 10years | | Withdrawn | 16 | 17 |
|  |  |  |  |  |  |  |  |  |  |  |  |  |  |  |  |  |  |  |  |  |  |  |  |  |  |  | PCR unadjusted LCF at day 42 | 15 | 42 |
|  |  |  |  |  |  |  |  |  |  |  |  |  |  |  |  |  |  |  |  |  |  |  |  |  |  |  | PCR adjusted LPF at day 42 | 49 | 75 |
|  |  |  |  |  |  |  |  |  |  |  |  |  |  |  |  |  |  |  |  |  |  |  |  |  |  |  | PCR adjusted LCF at day 42 | 1 | 3 |
|  | | | | | | | | | | | | | | | | | | | | | | | | | | | PCR unadjusted LPF at day 42 | 2 | 1 |
|  |  |  |  |  |  |  |  |  |  |  |  |  |  |  |  |  |  |  |  |  |  |  |  |  |  |  | Undetermined PCR | 6 | 8 |
|  |  |  |  |  |  |  |  |  |  |  |  |  |  |  |  |  |  |  |  |  |  |  |  |  |  |  | Parasite clearance at day 1 | 203 | 223 |
|  |  |  |  |  |  |  |  |  |  |  |  |  |  |  |  |  |  |  |  |  |  |  |  |  |  |  | Parasite clearance at day 2 | 38 | 56 |
|  |  |  |  |  |  |  |  |  |  |  |  |  |  |  |  |  |  |  |  |  |  |  |  |  |  |  | Parasite clearance at day 3 | 0 | 1 |
| 23 | | **Uwimana-2019-RWA** [81] | | | Open label, RCT | | | Health centers, September 2013 and December 2015 | | Moderate transmission | | | | | 42 days | | | | 269 | | | 267 | | | 1–14 years | | Withdrawn | 2 | 0 |
|  |  |  |  |  |  |  |  |  |  |  |  |  |  |  |  |  |  |  |  |  |  |  |  |  |  |  | ETF | 2 | 2 |
|  |  |  |  |  |  |  |  |  |  |  |  |  |  |  |  |  |  |  |  |  |  |  |  |  |  |  | PCR unadjusted LCF day 28 | 2 | 16 |
|  | | | | | | | | | | | | | | | | | | | | | | | | | | | PCR unadjusted LPF day 28 | 2 | 18 |
|  |  |  |  |  |  |  |  |  |  |  |  |  |  |  |  |  |  |  |  |  |  |  |  |  |  |  | PCR adjusted LCF day 28 | 1 | 2 |
|  |  |  |  |  |  |  |  |  |  |  |  |  |  |  |  |  |  |  |  |  |  |  |  |  |  |  | PCR adjusted LPF day 28 | 0 | 0 |
|  |  |  |  |  |  |  |  |  |  |  |  |  |  |  |  |  |  |  |  |  |  |  |  |  |  |  | PCR unadjusted LCF day 42 | 10 | 24 |
|  |  |  |  |  |  |  |  |  |  |  |  |  |  |  |  |  |  |  |  |  |  |  |  |  |  |  | PCR unadjusted LPF day 42 | 10 | 26 |
|  |  |  |  |  |  |  |  |  |  |  |  |  |  |  |  |  |  |  |  |  |  |  |  |  |  |  | PCR adjusted LCF day 42 | 1 | 3 |
|  |  |  |  |  |  |  |  |  |  |  |  |  |  |  |  |  |  |  |  |  |  |  |  |  |  |  | PCR adjusted LPF day 42 | 1 | 1 |
| 24 | | **Yeka-2019-UGA** [105] | | | | Single-blind RCT | | Health center and Hospital, October 2015-December, 2016 | High transmission | | | | | | 42 days | | | | 299 | | 300 | | | | 6-59 months | | Withdrawn | 11 | 10 |
|  |  |  |  |  |  |  |  |  |  |  |  |  |  |  |  |  |  |  |  |  |  |  |  |  |  |  | ETF | 0 | 0 |
|  |  |  |  |  |  |  |  |  |  |  |  |  |  |  |  |  |  |  |  |  |  |  |  |  |  |  | LCF | 32 | 50 |
|  | | | | | | | | | | | | | | | | | | | | | | | | | | | LPF | 43 | 85 |
|  |  |  |  |  |  |  |  |  |  |  |  |  |  |  |  |  |  |  |  |  |  |  |  |  |  |  | ACPR | 213 | 155 |
|  |  |  |  |  |  |  |  |  |  |  |  |  |  |  |  |  |  |  |  |  |  |  |  |  |  |  | Fever clearance at day 1^b^ | 208 | 231 |
|  |  |  |  |  |  |  |  |  |  |  |  |  |  |  |  |  |  |  |  |  |  |  |  |  |  |  | Fever clearance at day 2 | 71 | 73 |
|  |  |  |  |  |  |  |  |  |  |  |  |  |  |  |  |  |  |  |  |  |  |  |  |  |  |  | Fever clearance at day 3 | 31 | 18 |
|  |  |  |  |  |  |  |  |  |  |  |  |  |  |  |  |  |  |  |  |  |  |  |  |  |  |  | Parasite clearance at day 1 | 219 | 245 |
|  |  |  |  |  |  |  |  |  |  |  |  |  |  |  |  |  |  |  |  |  |  |  |  |  |  |  | Parasite clearance at day 2 | 22 | 34 |
|  |  |  |  |  |  |  |  |  |  |  |  |  |  |  |  |  |  |  |  |  |  |  |  |  |  |  | Parasite clearance at day 3 | 3 | 3 |
| 25 | | | **Gansane-2021-BNF** [73] | | | | Open label, RCT | Primary health facility and district hospital, November 2017 to September 2018 | | | Moderate to high transmission | | | | | | | 42 days | | 360 | | | | 360 | | 6-59 months | Withdrawn | 21 | 27 |
|  |  |  |  |  |  |  |  |  |  |  |  |  |  |  |  |  |  |  |  |  |  |  |  |  |  |  | Parasite clearance at day 3 | 6 | 1 |
|  |  |  |  |  |  |  |  |  |  |  |  |  |  |  |  |  |  |  |  |  |  |  |  |  |  |  | ETF | 6 | 1 |
|  | | | | | | | | | | | | | | | | | | | | | | | | | | | LCF at day 28 | 6 | 73 |
|  |  |  |  |  |  |  |  |  |  |  |  |  |  |  |  |  |  |  |  |  |  |  |  |  |  |  | LCF at day 28 | 10 | 104 |
|  |  |  |  |  |  |  |  |  |  |  |  |  |  |  |  |  |  |  |  |  |  |  |  |  |  |  | Recrudescence at day 28 | 7 | 54 |
|  |  |  |  |  |  |  |  |  |  |  |  |  |  |  |  |  |  |  |  |  |  |  |  |  |  |  | Re-infection at day 28 | 10 | 107 |
|  |  |  |  |  |  |  |  |  |  |  |  |  |  |  |  |  |  |  |  |  |  |  |  |  |  |  | Undetermined PCR at day 28 | 1 | 16 |
