# Supplementary material for: Efficacy of dihydroartemisinin-piperaquine versus artemether-lumefantrine for the treatment of uncomplicated Plasmodium falciparum malaria among children in Africa: a systematic review and meta-analysis of randomized control trials
Source: Malar J. 2021 Aug 12;20:340. doi: 10.1186/s12936-021-03873-1 (PMC8359548; doi:10.1186/s12936-021-03873-1)
Supplement: Supplementary file 6 — Additional file 6. Meta-regression of PCR-unadjusted treatment failure at day 28, association between malaria transmission intensity within the countries and treatment failure. [file 12936_2021_3873_MOESM6_ESM.docx]

Additional file S5: Meta- regression of PCR-unadjusted treatment failure at day 28, association between age of the children and treatment failure.
