# Supplementary material for: Efficacy of dihydroartemisinin-piperaquine versus artemether-lumefantrine for the treatment of uncomplicated Plasmodium falciparum malaria among children in Africa: a systematic review and meta-analysis of randomized control trials
Source: Malar J. 2021 Aug 12;20:340. doi: 10.1186/s12936-021-03873-1 (PMC8359548; doi:10.1186/s12936-021-03873-1)
Supplement: Supplementary file 7 — Additional file 7. Funnel plot of comparison: dihydroartemisinin-piperaquine versus artemether-lumefantrine for treatment of uncomplicated falciparum malaria in African children, outcome: PCR-unadjusted treatment failure at day 28. [file 12936_2021_3873_MOESM7_ESM.docx]

Additional file S6: Meta- regression of PCR-unadjusted treatment failure at day 28, association between malaria transmission intensity within the countries and treatment failure.
