# Supplementary material for: Efficacy of dihydroartemisinin-piperaquine versus artemether-lumefantrine for the treatment of uncomplicated Plasmodium falciparum malaria among children in Africa: a systematic review and meta-analysis of randomized control trials
Source: Malar J. 2021 Aug 12;20:340. doi: 10.1186/s12936-021-03873-1 (PMC8359548; doi:10.1186/s12936-021-03873-1)
Supplement: Supplementary file 8 — Additional file 8. Funnel plot of comparison: dihydroartemisinin-piperaquine versus artemether-lumefantrine for treatment of uncomplicated falciparum malaria in African children, outcome: PCR-adjusted treatment failure at day 28. [file 12936_2021_3873_MOESM8_ESM.docx]

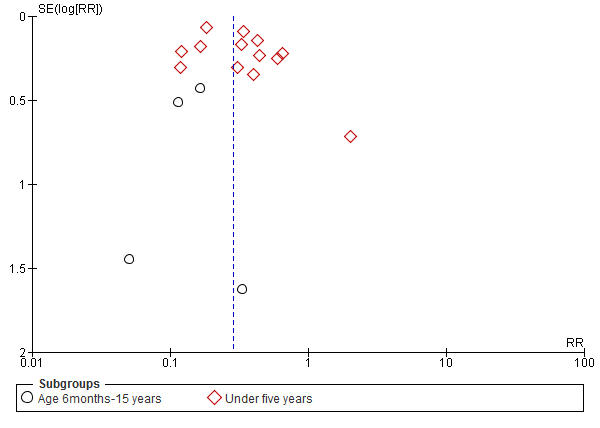


Additional file S7: Funnel plot of comparison: dihydroartemisinin-piperaquine versus artemether-lumefantrine for treatment of uncomplicated *plasmodium falciparum* malaria among children in Africa, outcome: PCR unadjusted treatment failure at day 28.
