# Supplementary material for: Efficacy of dihydroartemisinin-piperaquine versus artemether-lumefantrine for the treatment of uncomplicated Plasmodium falciparum malaria among children in Africa: a systematic review and meta-analysis of randomized control trials
Source: Malar J. 2021 Aug 12;20:340. doi: 10.1186/s12936-021-03873-1 (PMC8359548; doi:10.1186/s12936-021-03873-1)
Supplement: Supplementary file 11 — Additional file 11. Forest plot of comparison: dihydroartemisinin-piperaquine versus artemether-lumefantrine for treatment of uncomplicated falciparum malaria in African children, outcome: Parasite clearance day 2 and 3. [file 12936_2021_3873_MOESM11_ESM.docx]

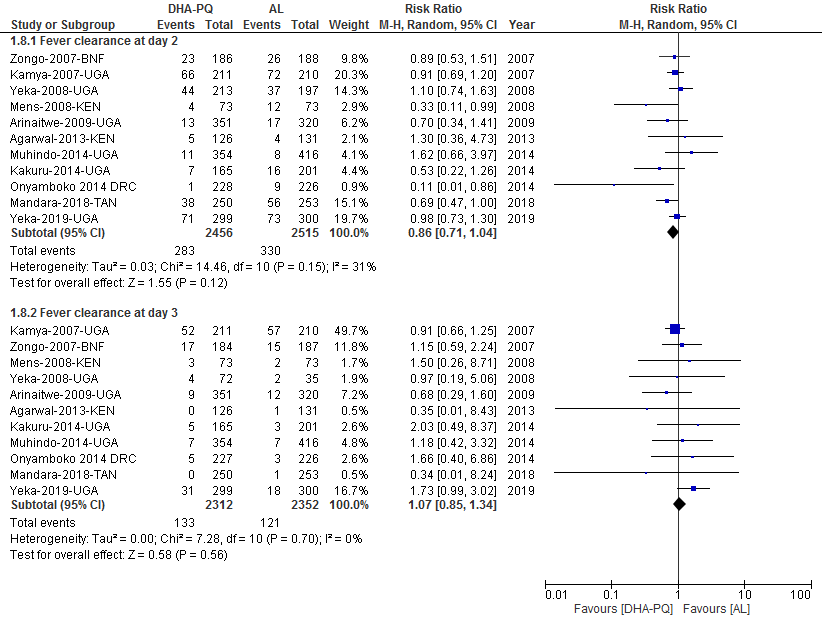


Additional file S10: Forest plot of comparison between dihydroartemisinin-piperaquine and artemether-lumefantrine for treatment of uncomplicated *plasmodium falciparum* malaria among children in Africa on fever clearance.
